# The second life of *Citrus bergamia*: bioavailability analysis of a new formulation using waste-based microencapsulation as a valuable source of bioactive compounds

**DOI:** 10.1007/s43440-025-00758-x

**Published:** 2025-07-25

**Authors:** Maria Serra, Roberta Macrì, Sonia Bonacci, Giovanna Ritorto, Sara Ussia, Saverio Nucera, Rosamaria Caminiti, Stefano Ruga, Carmen Altomare, Luigi Tucci, Giuseppe Trunfio, Donato Cosco, Antonio Procopio, Carolina Muscoli, Rocco Mollace, Vincenzo Mollace

**Affiliations:** 1 Pharmacology Laboratory, Institute of Research for Food Safety and Health IRC-FSH, Department of Health Sciences, University Magna Graecia of Catanzaro, Catanzaro, 88100 Italy; 2 Department of Health Sciences, University “Magna Graecia” of Catanzaro, Campus Universitario “S. Venuta”, Catanzaro, Italy; 3 Department of Experimental Medicine, University “Tor Vergata” of Rome, Rome, Italy; 4 Renato Dulbecco Institute, Lamezia Terme, Catanzaro, Italy

**Keywords:** Bioavailability, Bergamot polyphenolic fraction, Citrus albedo fibers, Oxidative stress, Inflammation, Nutraceuticals, Polyphenols, Encapsulation, Micronization, Green chemistry, Nutraceuticals

## Abstract

**Background:**

Polyphenols have garnered significant interest because of their potential health benefits, but their bioavailability is limited. According to recent studies, in vivo metabolites of phenol compounds may mediate their biological activity, potentially countering systemic oxidation and inflammation and therefore reducing multi-organ dysfunction associated with gut microbiota alterations. This pre-clinical study aims to characterize a novel formulation, enhancing metabolite bioavailability, ensuring long-term stability, and employing sustainable production methods. Our research provides the first evidence of the presence of these metabolites in the blood plasma of animals receiving different Bergamot polyphenols fraction (BPF) formulations.

**Methods:**

Male Sprague-Dawley were used throughout the study. The animals were subdivided into three groups of six animals each receiving 50 mg/kg of BPF standard (BPF), 50 mg/kg of Bergamot polyphenols fraction micronized (BPFmicro), and 50 mg/kgof Bergamot polyphenols fraction encapsulation (BPFencap), respectively, by oral gavage. Blood samples were collected, and plasma was prepared with a specific protocol and analysed for the presence of primary and secondary metabolites through ultra-high performance liquid chromatography-tandem mass spectrometry (UHPLC-MS/MS).

**Results:**

UHPLC-MS/MS analysis showed significantly higher plasma concentrations of naringin and its metabolites in the BPFencap group compared to the BPF standard and BPFmicro groups at all time points. In comparison to BPF, plasma Area Under Curve (AUC) analysis of metabolites revealed substantially elevated values for the BPFencap group and substantially reduced values for the BPFmicro group.

**Conclusion:**

While BPFmicro greatly increased bioavailability, the improvement was only temporary, highlighting a stability problem. The bioavailability and stability of metabolites are significantly improved over time by the new BPFencap formulation (micronized BPF in hybrid phospholipid systems with citrus albedo fibers).

**Graphical abstract:**

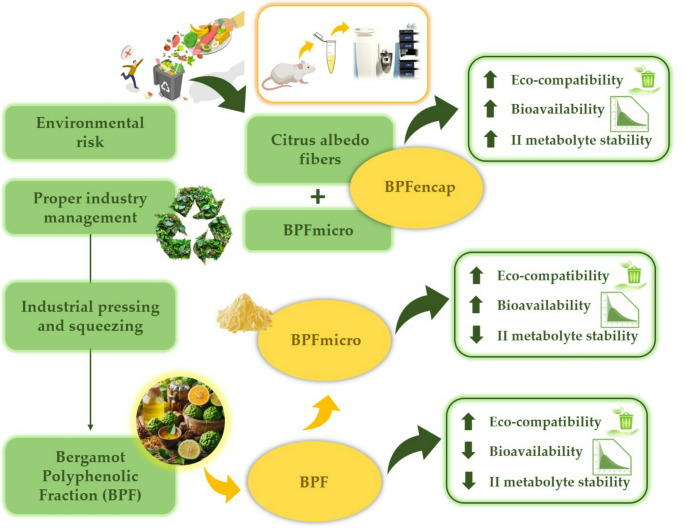

**Supplementary Information:**

The online version contains supplementary material available at 10.1007/s43440-025-00758-x.

## Introduction

Preparations of plant biomass or parts of it have been widely used in popular medicine since ancient times, and till today, the use of phytomedicines is widespread in most of the world’s population [1]. Indeed, as is commonly known, phytochemicals present in vegetables and fruits are vital in both health promotion and disease prevention, including cardiovascular diseases, cancers, as well as neurodegenerative disorders [2]. Among these compounds, flavonoids constitute one of the most ubiquitous groups of plant polyphenolics; due to the variety of their pharmacological activities in the mammalian body as powerful free radical scavengers and anti-allergic, anti-inflammatory, anti-microbial, and anti-cancer agents, they are more correctly referred to as nutraceuticals [3]. In view of this, over the last decade, some researchers have started to investigate the biological properties of Citrus fruits and juices, which are the principal dietary source of such important secondary metabolites [4]. In particular, the use of bergamot (*Citrus bergamia* Risso et Poiteau) in traditional medicine, forgotten for decades, is currently being rediscovered due to its characteristic pattern - almost unique - of polyphenolics, specifically flavonoids, present in its juice, albedo, flavedo, and fibers [5]. Bergamot is a common Italian citrus fruit, cultivated almost exclusively to produce essential oil from the fruit flavedo. The juice and albedo, on the other hand, and in contrast to those from other citrus fruits, are considered just a polluting waste of the essential oil production, with consequent economic and environmental disadvantages [6]. Furthermore, in the search for a re-evaluated and possibly more powerful pharmaceutical surrogate of bergamot juice and albedo, called “pastazzo”, a method to concentrate and exsiccate them was developed, producing a concentrated mixture of flavonoids, Bergamot Polyphenol Fraction (BPF) [7, 8]. BPF, patented by H&AD Srl, has a unique profile of flavonoids and glycosides which result clinically useful to help maintain healthy cholesterol, to counteract oxidative stress and suppress inflammation in endothelial cells, thus inhibiting plaque formation, and improving arterial responsiveness [9–12]. In addition, it has been observed that the combination of BPF extract and Albedo and pulp-derived micronized fibers (BMF) extract was able to significantly reduce glycaemic and lipemic profiles, with an important low-density lipoprotein (LDL) rearrangement, in rats fed with a high-fat diet [13]. Furthermore, the combination of BPF and BMF played a crucial role in counteracting microbiota alteration through the induction of short-chain fatty acid (SCFA) production and the proliferation of beneficial gut bacteria, such as lactobacillus and bifidobacterium [13]. Indeed, several studies showed that a diet rich in fiber and antioxidant bioactive compounds could play a crucial role in gut microbiota dysfunction and metabolic alterations [14].

The process is affected by dietary fibers, which lower polyphenol levels released into the upper digestive tract [15]. This process could lead to a higher concentration of polyphenols in the lower digestive tract, resulting in possible health advantages. Dietary fiber could influence the bioavailability of polyphenols within the digestive tract. Further research is needed to understand the impact of dietary fiber on polyphenol activity [16]. Dietary fiber is appealing not only for its nutritional value but also for its functional and technological properties [17]. Studies suggest that particle size affects how dietary fiber impacts the body. Compared to regular-sized particles, smaller dietary fiber particles might be more easily absorbed in the small intestine. Fiber particle size influences how much polyphenol is released and how accessible it is, thus necessitating additional research [16]. The micronization is used to decrease the particle size of the compound and increase the absorption and consequently the bioavailability. Indeed, in a study on the micronization of olive oil polyphenols, it was seen that decreasing the particle size of polyphenols increases bioaccessibility in the salivary and gastric phases of in vitro digestion [18]. In another study was evaluated the role of micronization in increasing orange flavanones bioavailability was evaluated, showing that hesperidin micronization (5.1 μm) increased flavanone’s bioavailability 2-fold compared to conventional hesperidin (32.8 μm) [19].

The release of colonic polyphenols, the quantity released, and the release mechanism are all important research questions. Studies explored the potential for colon fermentation to release and metabolize these compounds. Colonic polyphenol bioavailability in the lower gastrointestinal tract is determined by gut microbiota action [16, 20]. In addition, promising results from previous studies evaluated the pharmacokinetics of BPF extract and a new BPF formulation with PHYTOSOME^®^, a patented technology developed at Indena S.p.A. (Milan, Italy), showed the better absorption and pharmacokinetic profile of the new formulation compared to the standard formulation [21]. In an in vivo study, the presence of the main bioactive compounds of BPF was verified in the serum of mice [22]. Recently, our research group developed and patented a new way of formulating bioactive compounds using an innovative microencapsulation methodology, micronized citrus system (MCS), using Citrus albedo and phospholipids, supposed to enhance polyphenols bioavailability. Characterization of the metabolites and their potential functions is of great importance to practical applications. Currently, high-resolution mass spectrometry (HRMS) surpasses older liquid chromatography-mass spectrometry (LC-MS) systems in sensitivity and resolution, making it ideal for profiling and characterizing trace components in diverse samples. To determine BPF’s pharmacological activity, it’s essential to analyze its in vivo components after oral administration and identify the responsible chemicals. In light of all previous findings, this experimental study aimed to analyse, in wild-type male Sprague-Dawley rats, the bioavailability of secondary metabolites obtained from the main bioactive compounds administered through the innovative MCS hybrid formulation Bergamot polyphenols fraction encapsulation (BPFencap), containing BPF to increase polyphenol bioavailability to be used in finished nutraceutical products. The dose of 50 mg/kg has been chosen because this is the effective dose that results in a beneficial protective effect in several dysfunction models [23, 24]. This formulation will be compared with the BPF standard (BPF) and Bergamot polyphenols fraction micronized (BPFmicro).

## Materials and methods

### Standard and reagent

All chemicals and reagents were of analytical grade. LC/MS grade methanol (MeOH) was purchased from VWR BDH Chemicals (Milan, Italy) (CAS 67-56-1). Formic acid 99% (CAS 64-18-6) was purchased from Carlo Erba (Milan, Italy). Ultrapure water (18MΩ) was obtained by a-milli-Q purification system (Millipore, Bedford, MA, USA). Reference standards of naringin (PHL89739) and naringenin (PHL89738) were purchased from PhytoLab. Naringin-7-*O*-glucuronide (CAY-10388) was purchased from Cayman Chemical, and: Ethylenediaminetetraacetic acid (EDTA), 0.5M (A4892,0100), was purchased from PanReac Applichem. Solid phase extraction (SPE) columns, CHROMABOND HLB, 30 μm, 1mL/100 mg.

### BPF Preparation for in vivo study

*Citrus bergamia* Risso & Poiteau fruits were collected from plants located in a range of 90 Km from Bianco to Reggio Calabria, Italy.

BPF was prepared as previously described [7]. The harvested fruit was peeled, squeezed, and processed to yield bergamot juice, oil, and pastazzo. Water was used to extract polyphenols from minced bergamot processing byproduct, “pastazzo”; a pectolytic enzyme was then added for pectin digestion. The clarified extract, via ultrafiltration, was then processed using a polystyrene absorbing resin. Subsequently, a cationic resin bed eluted and stabilized the trapped polyphenols, returning them to their original acidic pH [7] (Figure S1).

BPF and BPFmicro were solubilized in Mill-Q water and administered to rats by gavage at 50 mg/kg. BPF hybrid system biocompatible was formulated according to a specific technique, microencapsulation citrus system (MCS) (submitted patent 6355PTIT by 3M&F Consulting srl) to decrease the particle size of BPF micro powder and encapsulated 50/50 (micronized Citrus albedo fiber/BPF micro) p/p, solubilized in MilliQ, administered by gavage 50 mg/kg, the quantitative content of polyphenols in BPFencap is about half of the other two formulations.

### Animals

Male Sprague-Dawley 3-month-old rats (Charles River, Milan, Italy) weighing 270–290 g were used throughout the study. The animals were subdivided into three groups of six animals each: BPF, BPFmicro, and BPFencap.The number of animals selected was adequate to achieve statistical significance (p < 0.05). All animals were housed and cared for in accordance with the Italian National Health Ministry Guidelines on Laboratory Animal Welfare, following the Italian regulations for the protection of animals used for experimental and other scientific purposes (D.L. 26/2014) as well as with the European Community guidelines. Each cage housed two rats, which were housed and maintained under controlled conditions of temperature (21°C) and humidity (60–65%) with a 12 h light/12 h dark cycle and allowed food ad libitum. The study was conducted according to the guidelines of the Declaration of Helsinki and approved by the local Ethics Committee (Calabrian Region Prot. 324: 12 October 2021), with the title” Pre-clinical and Clinical Study on the effect of BPF and/or albedo and pulp-derived micronized fibers (BMF) on gut microbioma and lipid metabolism”, PI: Prof. Vincenzo Mollace (Figure S2).

### Study design and treatment

**Fig. 1:**
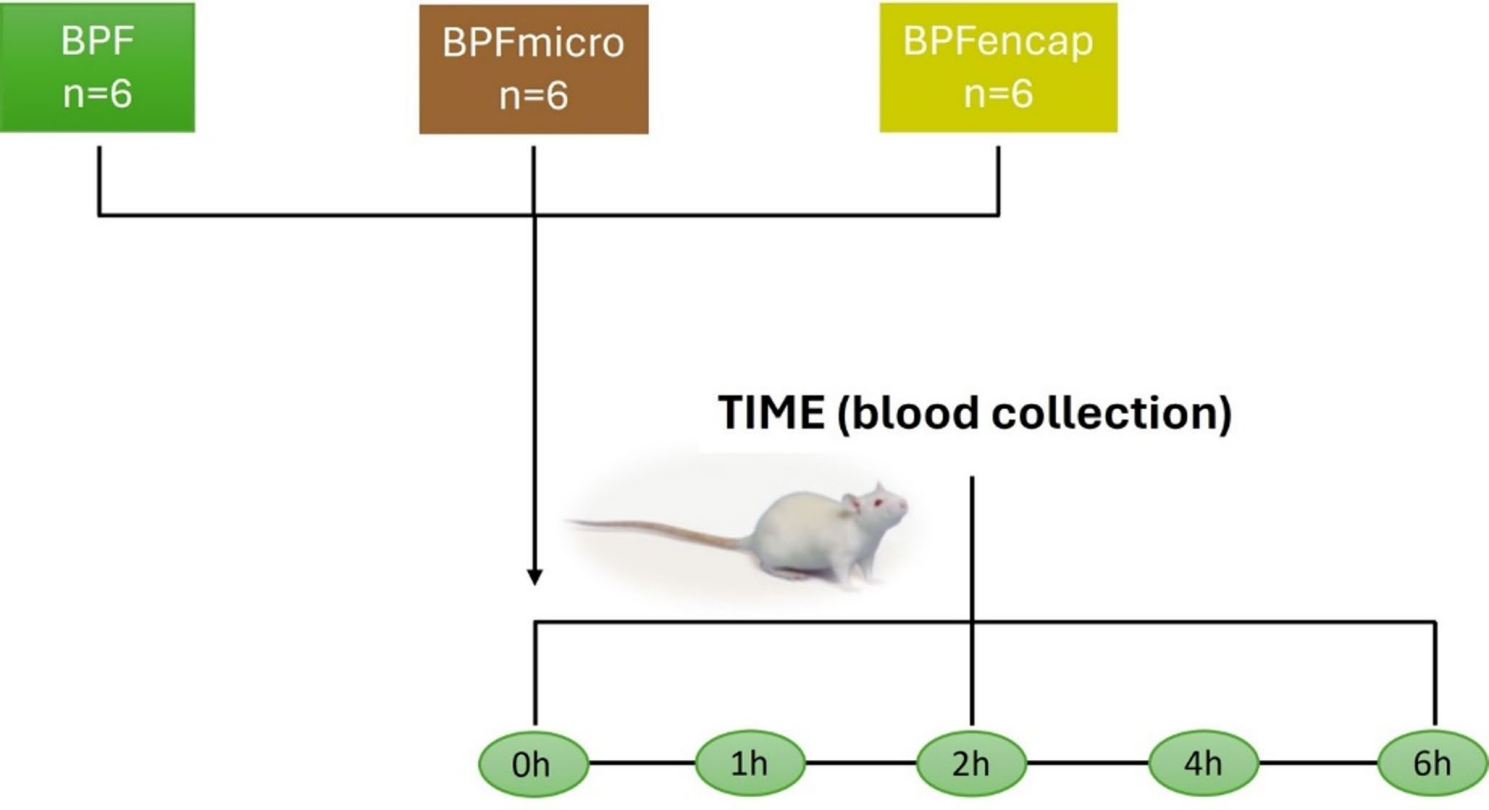
Study design: The animals were treated with BPF (*n* = 6), BPFmicro (*n* = 6), and BPFencap (*n* = 6), respectively. At time 0 and after 1, 2, 4, and 6 h of treatment, the blood was collected for bioavailability assay. BPF: Bergamot polyphenolic fraction; BPFmicro: Bergamot polyphenols fraction micronized; BPFencap: Bergamot polyphenols fraction encapsulation

The dosing solution of each treatment was freshly prepared and administered via an oral gavage at a single dose of 50 mg/kg. The animals had free access to water during the experiment. A series of blood samples was collected in 2 mL Eppendorf tubes with EDTA from the tail vein of each rat at 0, 1, 2, 4, and 6 h and stored at − 80 °C until analysis (Fig. 1).

### Blood collection and solid phase extraction (SPE)

After collecting blood samples in a 2 mL erppendorf tube, with 12 µL of EDTA 0.5 M, gently mixed, and chilled on ice. Once centrifuged at 3,000 rpm for 15 min, the supernatant (plasma) was transferred to a 0.5 mL Eppendorf tube and spun at 3,000 rpm for 15 s, stored at -80 °C before the analysis. Aliquots of plasma samples (400 µL) were placed in a 500 µL Eppendorf tube, and 50 µL of formic acid was added. The mixture was vortexed for 1.0 min and then loaded onto an activated SPE C_18_ column (the SPE C_18_ column was activated by 3 mL of methanol before loading the sample, and the excess was washed off with 3 mL of purified water). After rinsing with 3mL of purified water, the SPE column was eluted with mL1 mL of methanol, and the eluate was evaporated to dryness under a gentle stream of nitrogen. The residue was reconstituted in 150 µL methanol and centrifuged at 16,000 × g for 5 min; then, a 5µL aliquot was injected into the ultra-high performance liquid chromatography-tandem mass spectrometry.

(UHPLC-MS/MS) system for analysis.

### Analysis UHPLC/ESI- HRMS

The characterization of all samples was performed via ultra-high-performance liquid chromatography-electrospray ionization-high resolution mass spectrometry (UHPLC-ESI-HRMS). The system comprised a Dionex Ultimate 3000 RS (Thermo Scientific, Rodano, MI, Italy) interfaced to a Q-Exactive orbitrap mass spectrometer (Thermo Scientific, Rodano, MI, Italy) using an electrospray ionization source operating in negative mode. Using a Thermo Scientific Hypersil Gold C18 column (100 × 2.1 mm, 1.9 μm), chromatographic separation was achieved at 24 °C and 300 µl min-1 flow rate. The chromatographic column was prepared using a mobile phase of 98% solvent A (ultrapure water, 0.1% formic acid) and 2% solvent B (methanol). A linear increase in solvent B concentration was applied as follows: 2–23% (6 min), isocratic hold (5 min), 23–50% (7 min), 50–98% (5 min), isocratic hold (6 min), and a final return to 2% (6 min) with a 3 min isocratic hold.

A 5 µL sample was injected. Column wash and equilibration added up to a total run time of 38 min [25]. Negative polarity HESI was used with a resolving power of 70,000 (FWHM at m/z 200), an injection time of 100 ms, an automatic gain control target of 1*E6, and a scan range of 100–850 m/z. The MS/MS analysis had a resolution setting of 35,000; AGC target 1*E5; maximum IT 200ms; collision energy (stepped NCE): 25. A quadrupole isolation window of 1.6 m/z was used. The sheath gas and auxiliary gas comprised high-purity nitrogen at 30 and 10 arb units, respectively. Before each analysis, the instrument was calibrated with Thermo Fisher Scientific’s calibration solution. Characterization of compounds relied on their HRMS spectra, accurate masses, characteristic fragmentation patterns, and retention times. Xcalibur software version 4.1 managed instrument control and acquired the data. FreeStyle software, version 1.3, was used to analyze the data. The UHPLC-ESI-HRMS method’s sensitivity was determined by its lower limit of detection (LOD), lower limit of quantification (LOQ) and extraction recovery.

The LOD represented the lowest concentration discernable from the blank, unlike the LOQ, which was the lowest reliably measured analyte concentration [25]. Each molecule’s concentration was determined using its respective commercial analytical standard’s external calibration curve.

### Statistical analysis

GraphPad PRISM 9.3.1 (GraphPad Software, Inc., La Jolla, CA, USA) was used for data analysis. Data are expressed as mean ± S.D., with normality tested via the Shapiro–Wilk test. Normally distributed data underwent one-way ANOVA and Tukey’s multiple comparison test. Grouped analysis, with two-way ANOVA, has been used to analyze the time effects and the treatment effects. A p-value below 0.05 indicated statistical significance.

## Results

### Chemical characterization by UHPLC-ESI-HRMS analysis

**Fig. 2:**
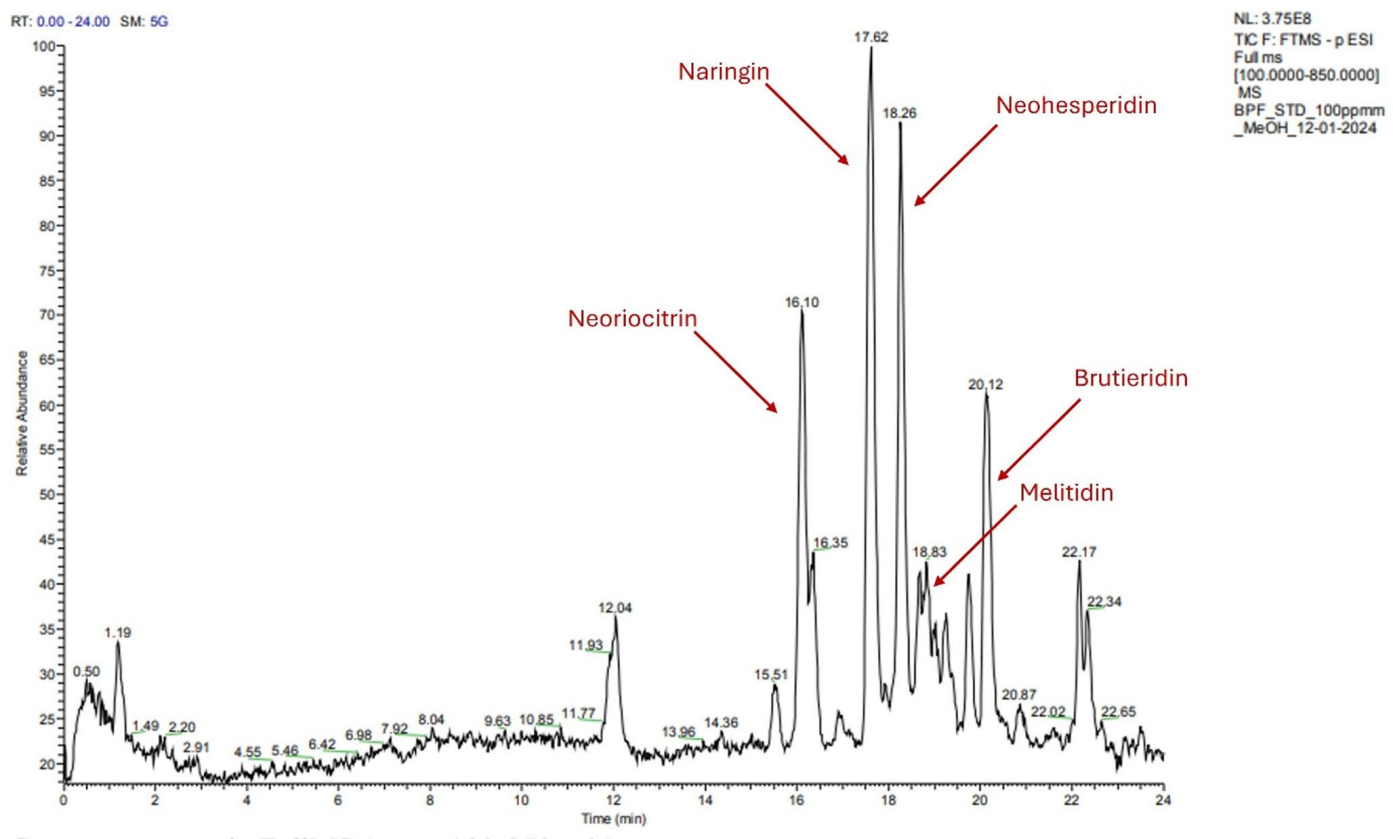
Full Scan chromatogram obtained by UHPLC-ESI-HRMS [M-H]^−^ of BPF extract (range 100–850 m/z). BPF: Bergamot polyphenolic fraction. UHPLC-ESI-HRMS: ultra-high-performance liquid chromatography-electrospray ionization-high resolution mass spectrometry

A comprehensive tentative characterization of BPF extract (Fig. 2) and plasma samples was carried out by UHPLC-ESI-HRMS analysis by comparing the mass data with literature data, considering the experimental *m/z* and the fragmentation patterns (Figure S3 and S4).

**Fig. 3:**
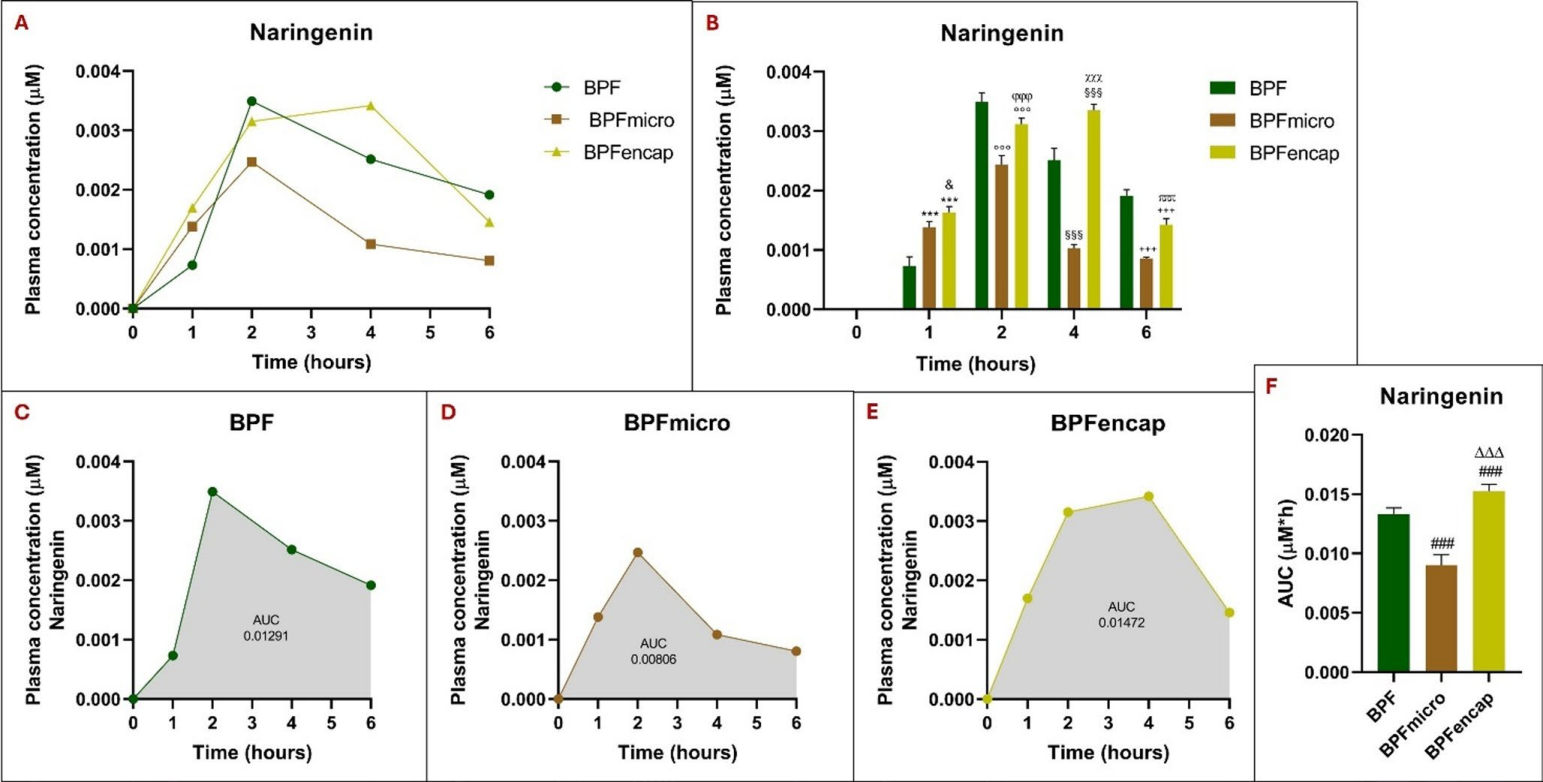
Naringenin plasma concentrations at 0-1-2-4-6 h in rats (*n* = 6 for each group) following oral administration of 50 mg/kg of BPF, BPFmicro and BPFencap, analyzed by ultra-high-performance liquid chromatography-electrospray ionization-high resolution mass spectrometry after solid phase extraction. Data were shown as mean ± SD (*n* = 6). (**A**) comparison of naringenin plasma concentrations across three groups; (**B**) Histogram of the comparison analysis of naringenin concentration over time in the three groups at 0-1-2-4-6 h: ***:*p* < 0.001 vs. BPF t1; °°°:*p* < 0.001 vs. BPF t2; §§§:*p* < 0.001 vs. BPF t4; +++: *p* < 0.001 vs. BPF t6; &: *p* < 0.05 vs. BPFmicro t1; ϕϕϕ:*p* < 0.001 vs. BPF micro t2; χχχ: *p* < 0.001 vs. BPFmicro t4; πππ:*p* < 0.001 vs. BPFmicro t6. (**C**), (**D**), (**E**) Naringenin’s plasma concentration time curves (AUC) in BPF, BPFmicro, and BPFencap groups. (**F**) Histogram of the comparison analysis of Naringenin AUC in the three groups: ###: *p* < 0.001 vs. BPF; ΔΔΔ: *p* < 0.001 vs. BPFmicro. All statistical analyses were performed using one-way ANOVA and Tukey’s multiple comparison test; Grouped analysis, with two-way ANOVA, has been used to compare within each row. BPF: Bergamot polyphenolic fraction; BPFmicro: Bergamot polyphenols fraction micronized; BPFencap: Bergamot polyphenols fraction encapsulation

Table 1 shows the results of metabolite identification. Both phase 1 and phase 2 metabolites are identified in rat plasma samples. Two metabolites (compounds 1 and 3), which yielded deprotonated molecular ions of *m*/*z* 447, were detected in all samples (shown in Table 1) and identified as naringenin-*O*- glucuronides. In MS^2^ spectra, characteristic fragments at *m/z* 271, originating by the loss of the glucuronyl unit, and/or at *m/z* 175, originating by the loss of aglycon naringenin, were found [26]. Similar to the fragmentation pattern of naringenin glucuronides, the hesperetin glucuronides produced two major characteristic ions via the loss of the glucuronyl moiety (giving an ion of *m*/*z* 301) or the aglycon hesperetin (giving an ion of *m*/*z* 175), respectively [27]. Naringin (*m/z* 579.1721*)* was found in trace amounts in the rat plasma samples and, as expected in the gastrointestinal tract, microbial enzymes could eliminate the glycoside of naringin to yield its aglycone naringenin (*m/z* 271). The presence of trace naringin in rat plasma after oral administration of BPF could be due to a gradual hydrolysis of naringin to naringenin to absorption [28]. In another study, it was shown that the high degree of transformation of naringin into phenolic acids in the dog’s alimentary tract may be one of the reasons why the bioavailability of naringin was poor [29]. Naringenin was identified based on its fragmentation pattern; the fragment ions of *m/z* 151 and 119 from *m/z* 271 were yielded through retro-Diels-Alder (RDA) reactions by breaking two C-C bonds of the C-ring [30]. In rats, naringenin was metabolized in the liver and converted into glucuronide intermediates. This liver metabolism may limit the bioavailability of naringin in plasma in vivo. Regarding the glucuronate metabolites hesperetin-7-*O*-glucuronide and hesperetin-5-*O*-glucuronide could be derivatives from neohesperidine. In these molecules, the fragment ions m/z 301 were generated due to deglycosylation. The fragment ions at *m/z* 301 could be hesperetin, in trace amounts in the plasma samples; this aglycon is rapidly metabolized in the body, making it difficult to maintain a higher blood concentration [31]. For this reason, its conjugates are the circulating metabolites like hesperetin-7-*O*-glucuronide and hesperetin-5-*O*-glucuronide (Figs. 2 and 3).

**Table 1:** Compounds identified in rat plasma by UHPLC-ESI-HRMS

Compound	R.T. (min)	[M-H]^−^ (m/z)	LC/HESI-HRMS^2^ (m/z)	Tentative identification
1	17.01	447.0946.	271.0616; 175.0241; 151.0027; 113.0233; 85.0282	Naringenin-5-*O* -glucuronide
2	17.95	477.1768	301.0720; 175.0240; 113.0232; 85.0282	Hesperetin-7*-O*-glucuronide
3	17.38	447.0936	271.0613; 175.0240; 151.0026; 113.0232; 85.0282	Naringenin-4-*O*-glucuronide
4	17.50	579.1721	459.1136; 271.0614; 151.0027; 119.0492	Naringin
5	17.59	477.1765.	301.1448; 175.0240; 113.0232; 85.0282	Hesperetin-5-*O*-glucuronide
6	20.94	301.0720	286.0485; 257.0483; 151.0027; 242.0583; 164.9263;	Hesperetin
7	20.56	271.0616	242.9757; 151.0027; 119.0490; 93.0333	Naringenin

UHPLC-ESI-HRMS: ultra-high-performance liquid chromatography-electrospray ionization-high resolution mass spectrometry

**Fig. 4:**
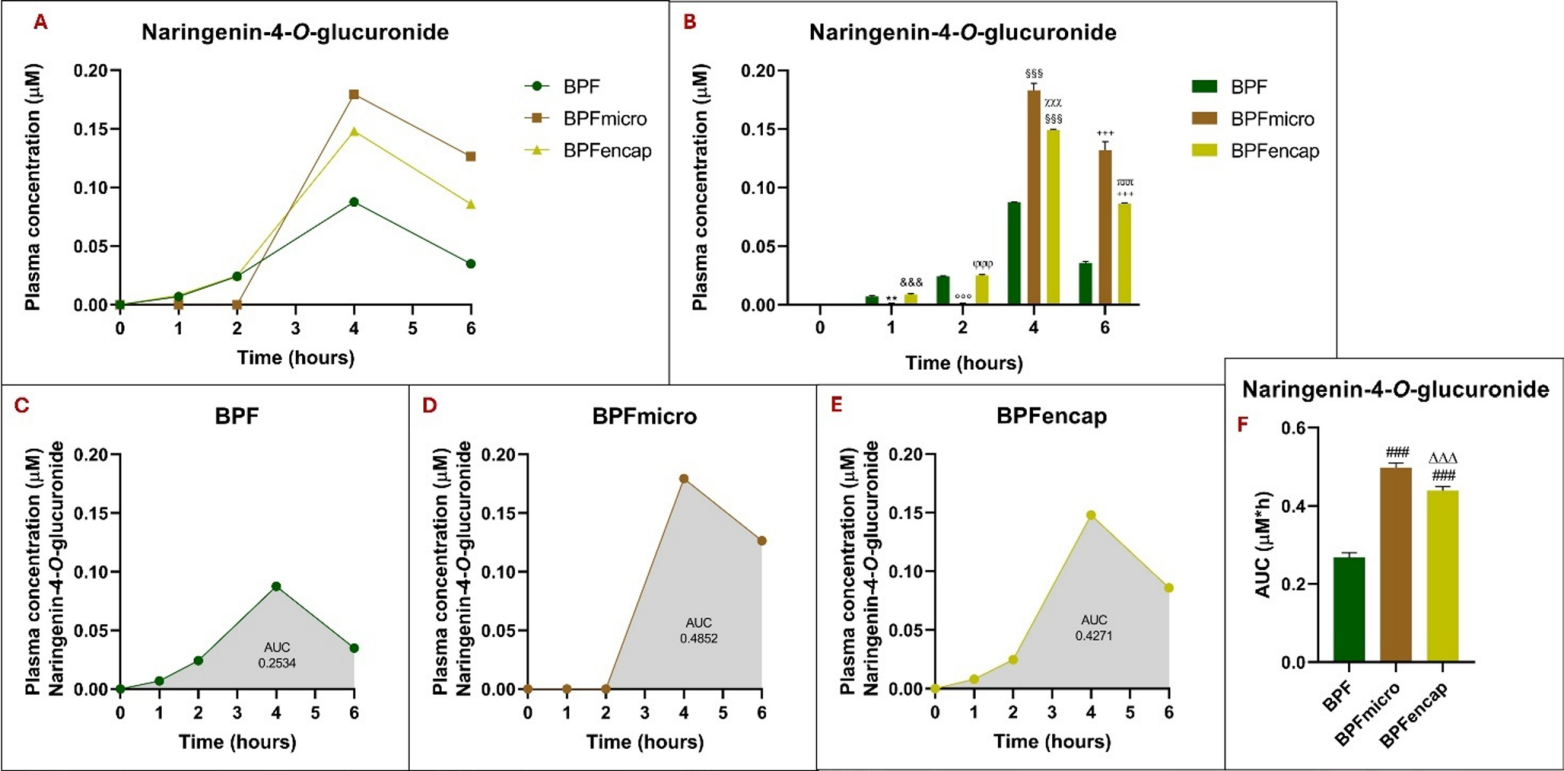
Naringenin-4-*O*-glucuronide plasma concentrations at 0-1-2-4-6 h in rats (*n* = 6 for each group) following oral administration of 50 mg/kg of BPF, BPFmicro and BPFencap, analyzed by ultra-high-performance liquid chromatography-electrospray ionization-high resolution mass spectrometry after solid phase extraction. (**A**) comparison of naringenin-4-O-glucuronide plasma concentrations across three groups; (**B**) Histogram of the comparison analysis of naringenin-4-*O*-glucuronide concentration over time in the three groups at different times (to, t1, t2, t4, t6): **:*p* < 0.001 vs. BPF t1; °°°:*p* < 0.001 vs. BPF t2; §§§:*p* < 0.001 vs. BPF t4; +++: *p* < 0.001 vs. BPF t6; &&&: *p* < 0.001 vs. BPFmicro t1; ϕϕϕ:*p* < 0.001 vs. BPF micro t2; χχχ: *p* < 0.001 vs. BPFmicro t4; πππ:*p* < 0.001 vs. BPFmicro t6. (**C**), (**D**), (**E**) naringenin-4-*O*-glucuronide’s plasma concentration time curves (AUC) in BPF, BPFmicro, and BPFencap groups. (**F**) Histogram of the comparison analysis of naringenin-4-*O*-glucuronide AUC in the three groups: ###: *p* < 0.001 vs. BPF; ΔΔΔ: *p* < 0.001 vs. BPFmicro. All statistical analyses were performed using one-way ANOVA and Tukey’s multiple comparison test; grouped analysis, with two-way ANOVA, has been used to compare within each row. BPF: Bergamot polyphenolic fraction; BPFmicro: Bergamot polyphenols fraction micronized; BPFencap: Bergamot polyphenols fraction encapsulation

The concentrations of naringenin (271.0616 *m/z*) were obtained using the calibration curve of the corresponding reference standards (r^2^ = 0.9912) in a range between 0.00018 and 0.03673 µM (Fig. 4). LOQ and LOD were estimated, respectively, 0.00018 µM and 0.00005 µM. The concentration of naringin (579.1721 *m/z)* was obtained using the calibration curve of the corresponding reference standards (r^2^ = 0.9934) in a range between 0.01722 µM -1.72253 µM. LOQ and LOD were estimated, respectively 0.01722 µM and 0.00517 µM and naringin-5-*O*-glucuronide (447.0946 *m/z*), hesperetin-7-*O*-glucuronide (477.1768 *m/z*), naringenin-4-*O*-glucuronide (447.0936 *m/z*), hesperetin-5-*O*-glucuronide (477.1765 *m/z*) were obtained respect to a calibration curve of naringenin-5-*O*-glucuronide (447.0946 *m/z*) reference standards (r^2^ = 0.9987) in a range between 0.11150 µM − 11.15075 µM. LOQ and LOD were estimated respectively 0.11150 µM and 0.033483 µM (Table 2). A specification sheet of Calibration curve of corresponding reference standards is shown in the Supplementary Materials (Figure S5).

**Table 2:** Analytical parameters: correlation coeffients, limit of detection (LOD) and limit of quantification (LOQ)

Compound	[M-H]^−^ (m/z)	*R* ^2^	LOD	LOQ
Naringenin	271.0616	0.9912	0.00005 µM	0.00018 µM
Naringenin-5-*O*-glucuronide	447.094	0.9987	0.033483 µM	0.11150 µM
Naringin	579.1721	0.9934	0.00517 µM	0.01722 µM

### Bioavailability of BPF metabolites in different formulations

Naringenin plasma concentration showed a significant variability at different times in BPFmicro and BPFencap concerning BPF standard (BPF) (Fig. 3). Of note, the naringenin plasma concentration peaks at 2 h post-treatment in animals receiving either BPF or BPFmicro; however, BPF standard achieves a significantly higher peak than BPFmicro. Concerning BPFencap, naringenin plasma levels are notably higher after 2 h, but still significantly less than with BPF. In BPFencap, the naringenin plasma peak is reached 4 h post-treatment, demonstrating a significant increase relative to standard BPF: *p* < 0.001). Six hours post-treatment, naringenin levels dropped across all three groups; however, the standard BPF group maintained the highest concentration. For a two-way ANOVA, the statistical results showed the following pattern: treatment effect (F_2,30_ = 205.9; *p* < 0.001), time effect (F_4,30_ = 1003; *p* < 0.001), interaction (F_8,30_ = 87.63; *p* < 0.001) (Table 3).

**Table 3:** Results of a two-way ANOVA for naringenin concentration in plasma, measured by UHPLC-ESI-HRMS

Independent variables	F-value	*p*-value
Time effect	F_4,30_ = 1003	*p* < 0.001
Treatment effect	F_2,30_ = 205.9	*p* < 0.001
Interaction	F_8,30_ = 87.63	*p* < 0.001

UHPLC-ESI-HRMS: ultra-high-performance liquid chromatography-electrospray ionization-high resolution mass spectrometry

Area Under Curve (AUC) analysis revealed a significantly higher naringenin concentration in the BPFencap group than in the standard BPF group (one-way ANOVA results: F_2,15_= 129.7; *p* < 0.001). The BPFmicro group shows the lowest AUC, with significantly lower total concentration than standard BPF and BPFencap (*p* < 0.001).

**Fig. 5:**
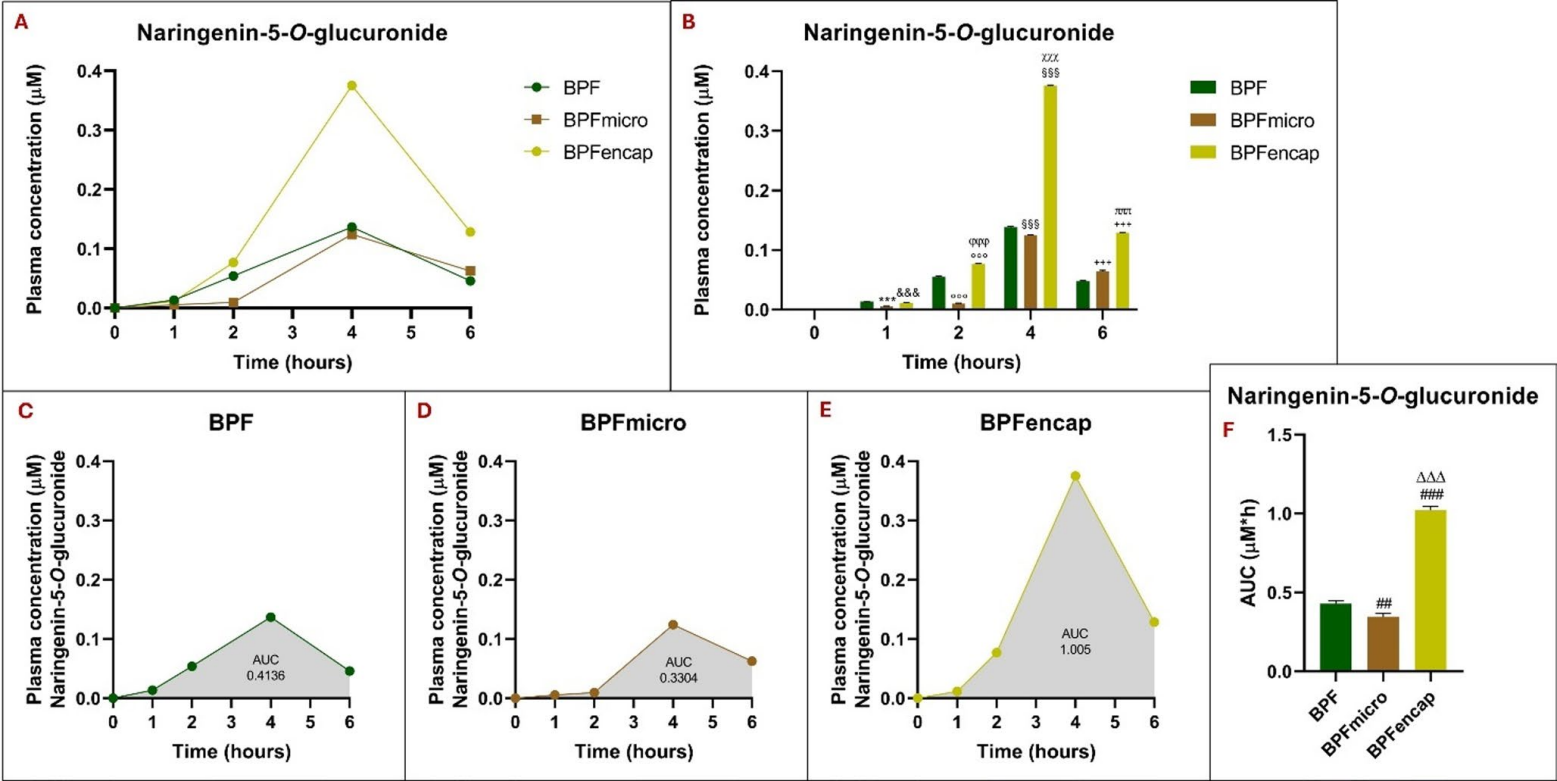
Naringenin-5-*O*-glucuronide plasma concentrations at 0-1-2-4-6 h in rats (*n* = 6 for each group) following oral administration of 50 mg/kg of BPF, BPFmicro and BPFencap, analyzed by ultra-high-performance liquid chromatography-electrospray ionization-high resolution mass spectrometry after solid phase extraction. (**A**) comparison of naringenin-5-*O*-glucuronide plasma concentrations across three groups; (**B**) Histogram of the comparison analysis of naringenin-5-*O*-glucuronide concentration over time in the three groups at different times (to, t1, t2, t4, t6): ***:*p* < 0.001 vs. BPF t1; °°°:*p* < 0.001 vs. BPF t2; §§§:*p* < 0.001 vs. BPF t4; +++: *p* < 0.001 vs. BPF t6; &&&: *p* < 0.001 vs. BPFmicro t1; ϕϕϕ:*p* < 0.001 vs. BPF micro t2; χχχ: *p* < 0.001 vs. BPFmicro t4; πππ:*p* < 0.001 vs. BPFmicro t6. (**C**), (**D**), (**E**) naringenin-5-*O*-glucuronide’s plasma concentration time curves (AUC) in BPF, BPFmicro, and BPFencap groups. (**F**) Histogram of the comparison analysis of naringenin-5-*O*-glucuronide AUC in the three groups: ##: *p* < 0.001 vs. BPF; ΔΔΔ: *p* < 0.001 vs. BPFmicro. All statistical analyses were performed using one-way ANOVA and Tukey’s multiple comparison test; grouped analysis, with two-way ANOVA, has been used to compare within each row. BPF: Bergamot polyphenolic fraction; BPFmicro: Bergamot polyphenols fraction micronized; BPFencap: Bergamot polyphenols fraction encapsulation

Secondary metabolite plasma concentrations were also assessed (Figs. 4 and 5). Specifically, examining naringenin-4-*O*-glucuronide concentration curves (Fig. 4) reveals near-zero plasma concentrations at 1 h and 2 h post-treatment in the BPFmicro group, contrasting with increases observed in the BPF and BPFencap groups. Plasma peaks were observed in all three groups four hours post-treatment. Compared to standard BPF, BPFmicro and BPFencap have considerably greater naringenin-4-*O*-glucuronide concentrations; BPFmicro-treated animals reach the highest plasma levels. After 6 h of treatment, naringenin-4-*O*-glucuronide concentrations in BPFmicro and BPFencap, although decreased, remain significantly higher with respect to BPF standard (*p* < 0.001). For a two-way ANOVA, the statistical results showed the following pattern: treatment effect (F_2,30_ = 689.1; *p* < 0.001), time effect (F 4, 30 = 5594; *p* < 0.001), interaction (F_8,30_ = 437.3; *p* < 0.001) (Table 4).

**Table 4:** Results of a two-way ANOVA for naringenin-4-*O*-glucuronide concentration in plasma of, measured by UHPLC-ESI-HRMS

Independent variables	F-value	*p*-value
Time effect	F_4,30_ = 5594	*p* < 0.001
Treatment effect	F_2,30_ = 689.1	*p* < 0.001
Interaction	F_8,30_ = 437.3	*p* < 0.001

UHPLC-ESI-HRMS: ultra-high-performance liquid chromatography-electrospray ionization-high resolution mass spectrometry

The BPFmicro and BPFencap groups exhibited significantly increased (one-way ANOVA results: F_2,15_=689.3; *p* < 0.001;) naringenin-4-*O*-glucuronide AUC values compared to the BPF group, following the trend over time. The highest significant increase is seen in the BPFmicro group (*p* < 0.001 vs. BPF and BPFencap).

**Fig. 6:**
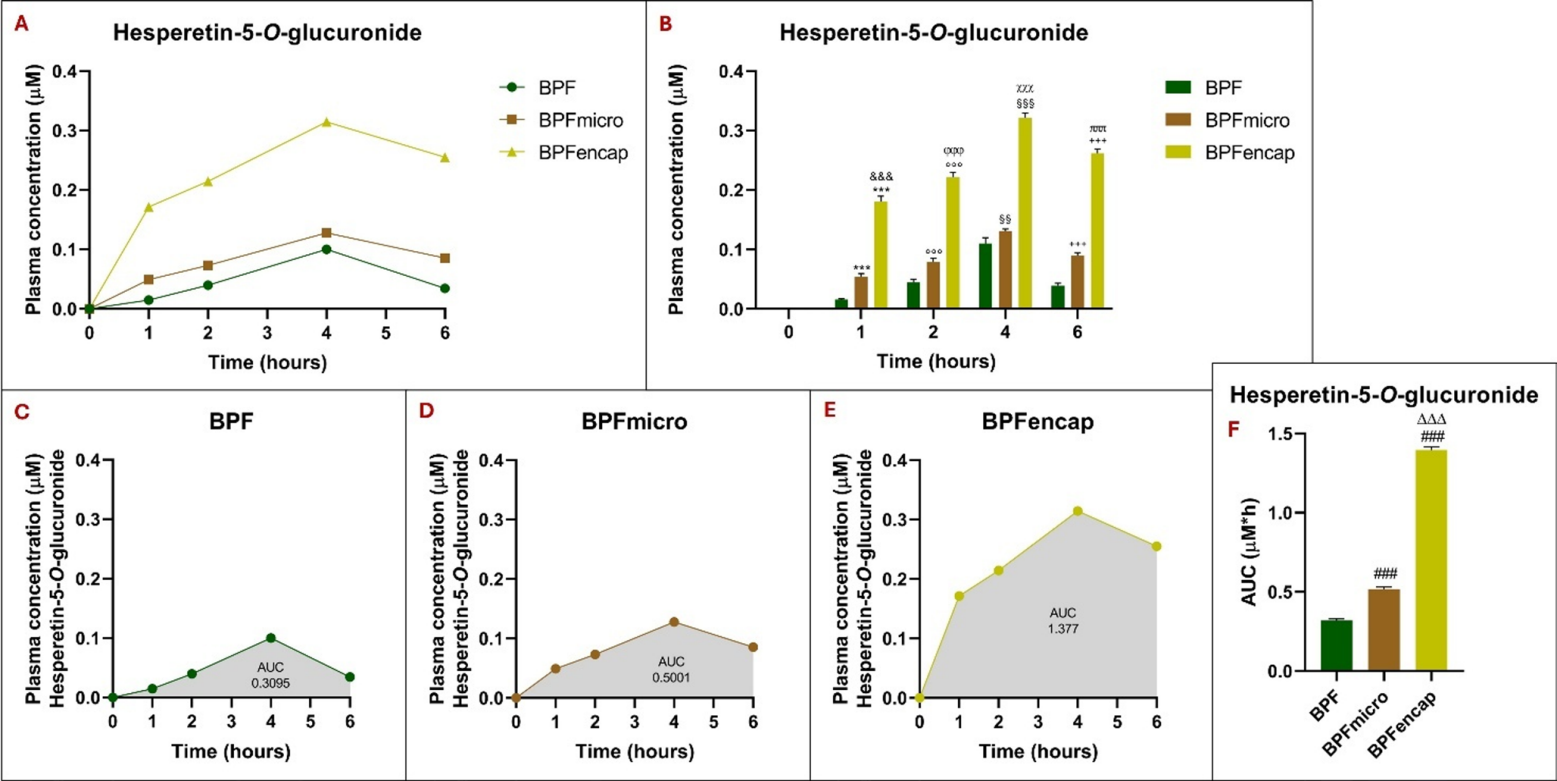
Hesperetin-5-*O*-glucuronide plasma concentrations at 0-1-2-4-6 h in rats (*n* = 6 for each rat) following oral administration of 50 mg/kg of BPF, BPFmicro and BPFencap, analyzed by ultra-high-performance liquid chromatography-electrospray ionization-high resolution mass spectrometry after solid phase extraction. (**A**) comparison of hesperetin-5-*O*-glucuronide plasma concentrations across three groups; (**B**) Histogram of the comparison analysis of hesperetin-5-*O*-glucuronide concentration over time in the three groups at different times (to, t1, t2, t4, t6): ***:*p* < 0.001 vs. BPF t1; °°°:*p* < 0.001 vs. BPF t2; §§:*p* < 0.01 vs. BPF t4; §§§:*p* < 0.001 vs. BPF t4; +++: *p* < 0.001 vs. BPF t6; &&&: *p* < 0.001 vs. BPFmicro t1; ϕϕϕ:*p* < 0.001 vs. BPF micro t2; χχχ: *p* < 0.001 vs. BPFmicro t4; πππ:*p* < 0.001 vs. BPFmicro t6. (**C**), (**D**), (**E**) hesperetin-5-*O*-glucuronide’s plasma concentration time curves (AUC) in BPF, BPFmicro, and BPFencap groups. (**F**) Histogram of the comparison analysis of hesperetin-5-*O*-glucuronide AUC in the three groups: ###: *p* < 0.001 vs. BPF; ΔΔΔ:*p* < 0.001 vs. BPFmicro. All statistical analyses were performed using one-way ANOVA and Tukey’s multiple comparison test; grouped analysis, with two-way ANOVA, has been used to compare within each row. BPF: Bergamot polyphenolic fraction; BPFmicro: Bergamot polyphenols fraction micronized; BPFencap: Bergamot polyphenols fraction encapsulation

A significant increase in naringenin-5-*O*-glucuronide plasma concentrations was observed in the BPFencap group, for BPF standard and BPFmicro (*p* < 0.001, respectively), contrasting the prior trend (Fig. 6). Metabolite concentrations are greater in the standard BPF group at the one-hour post-dose mark. But after two hours, the BPFencap group’s concentration increases markedly compared to standard BPF (*p* < 0.001) and BPFmicro (*p* < 0.001), reaching its highest point at four hours post-administration. A reduction in concentration is observed in the BPFencap group at 6 h, however, it remains markedly higher than the standard BPF (*p* < 0.001) and BPFmicro (*p* < 0.001). All three groups reach their peak at 4 h, however, only at 6 h does the BPF micro group show a higher plasma concentration of naringenin-5-o-glucuronide than the standard BPF (*p* < 0.001). For a two-way ANOVA, the statistical results showed the following pattern: treatment effect (F_2,30_ = 28346; *p* < 0.001), time effect (F_4,30_ = 70081; *p* < 0.001), interaction (F_8,30_ = 11068; *p* < 0.001) (Table 5).

**Table 5:** Results of a two-way ANOVA for naringenin-5-*O*-glucuronide naringenin concentration in plasma, measured by UHPLC-ESI-HRMS

Independent variables	F-value	*p*-value
Time effect	F_4,30_ = 70,081	*p* < 0.001
Treatment effect	F_2,30_ = 28,346	*p* < 0.001
Interaction	F_8,30_ = 11,068	*p* < 0.001

UHPLC-ESI-HRMS: ultra-high-performance liquid chromatography-electrospray ionization-high resolution mass spectrometry

AUC analysis of naringenin-5-*O*-glucuronide reveals a significantly greater increase in the BPFencap group versus the BPF group over time (one-way ANOVA results: F_2,15_=1982; *p* < 0.001). Compared to standard BPF and BPFencap, the BPFmicro group shows a significantly lower AUC (*p* < 0.01 and *p* < 0.001, respectively).

In a very interesting and noteworthy way, analysis of the plasma concentration of the secondary metabolite hesperetin-5-*O*-glucuronide reveals a significant increase of the latter at all experimental times analysed, in the BPFencap group compared with the standard BPF group (*p* < 0.001) and BPFmicro (*p* < 0.001) (Fig. 6).

Compared to the BPF group, the BPFmicro group also had a significantly higher metabolite level (*p* < 0.01 and *p* < 0.001), although less intense, at all experimental time points. All three groups reach their peak at 4 h after administration.

For a two-way ANOVA, the statistical results showed the following pattern: treatment effect (F_2,30_ = 2996; *p* < 0.001), time effect (F_4,30_ = 1259; *p* < 0.001), interaction (F_8,30_ = 206.6; *p* < 0.001) (Table 6).

**Table 6:** Results of a two-way ANOVA of hesperetin-5-*O*-glucuronide concentration in plasma, measured by UHPLC-ESI-HRMS

Independent variables	F-value	*p*-value
Time effect	F_4,30_ = 1259	*p* < 0.001
Treatment effect	F_2,30_ = 2996	*p* < 0.001
Interaction	F_8,30_ = 206.6	*p* < 0.001

UHPLC-ESI-HRMS: ultra-high-performance liquid chromatography-electrospray ionization-high resolution mass spectrometry

AUC analysis of hesperetin-5-*O*-glucuronide reveals significantly higher levels in both BPFmicro and BPFencap groups versus the BPF group (*p* < 0.001), with BPFencap showing a significantly greater increase concerning BPFmicro (one-way ANOVA results: F_2,15_=8970; *p* < 0.001).

**Fig. 7:**
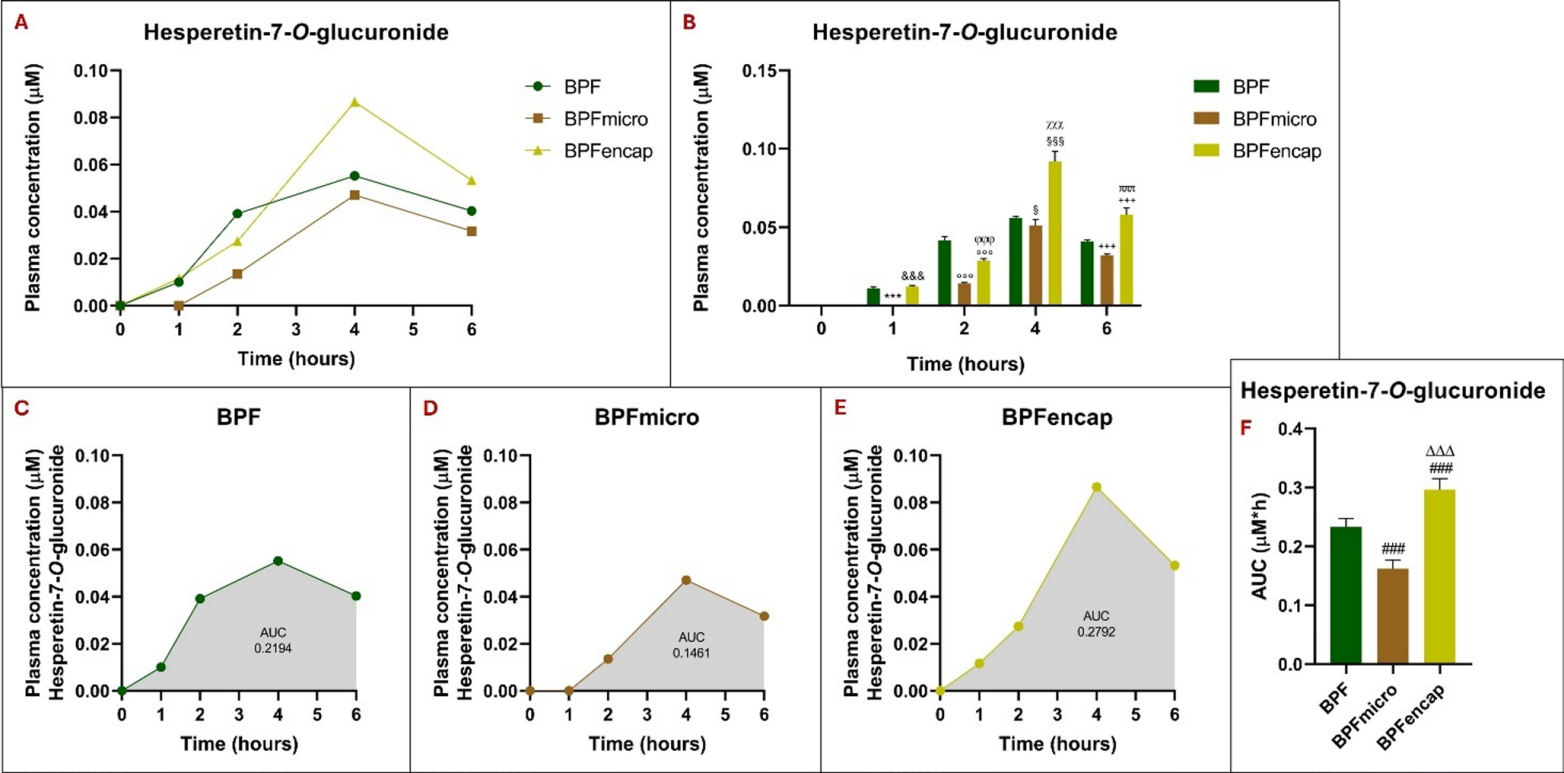
Hesperetin-7-*O*-glucuronide plasma concentrations at 0-1-2-4-6 h in rats (*n* = 6 for each group) following oral administration of 50 mg/kg of BPF, BPFmicro and BPFencap, analyzed by ultra-high-performance liquid chromatography-electrospray ionization-high resolution mass spectrometry after solid phase extraction. (**A**) comparison of hesperetin-7-*O*-glucuronide plasma concentrations across three groups; (**B**) Histogram of the comparison analysis of hesperetin-7-*O*-glucuronide concentration over time in the three groups at different times (to, t1, t2, t4, t6): ***:*p* < 0.001 vs. BPF t1; °°°:*p* < 0.001 vs. BPF t2; §:*p* < 0.05 vs. BPF t4; §§§:*p* < 0.001 vs. BPF t4; +++: *p* < 0.001 vs. BPF t6; &&&: *p* < 0.001 vs. BPFmicro t1; ϕϕϕ:*p* < 0.001 vs. BPF micro t2; χχχ: *p* < 0.001 vs. BPFmicro t4; πππ:*p* < 0.001 vs. BPFmicro t6. (**C**), (**D**), (**E**) hesperetin-7-*O*-glucuronide’s plasma concentration time curves (AUC) in BPF, BPFmicro, and BPFencap groups. (**F**) Histogram of the comparison analysis of hesperetin-7-*O*-glucuronide AUC in the three groups: ###: *p* < 0.001 vs. BPF; ΔΔΔ:*p* < 0.001 vs. BPFmicro. All statistical analyses were performed using one-way ANOVA and Tukey’s multiple comparison test; grouped analysis, with two-way ANOVA, has been used to compare within each row. BPF: Bergamot polyphenolic fraction; BPFmicro: Bergamot polyphenols fraction micronized; BPFencap: Bergamot polyphenols fraction encapsulation

Finally, analysis of the plasma concentration of hesperetin-7-*O*-glucuronide shows a similar trend to that of hesperetin-5-*O*-glucuronide in terms of the significant increase in concentration, at all experimental times, for the BPFencap group compared with the BPF group (*p* < 0.001) and BPFmicro (*p* < 0.001) (Fig. 7).

Conversely, the BPFmicro group exhibited a substantial decrease in all experimental times except the 4-hour mark (*p* < 0.05) when compared to the standard BPF (*p* < 0.001).

For a two-way ANOVA, the statistical results showed the following pattern: treatment effect (F_2,30_ = 229.3; *p* < 0.001), time effect (F_4,30_ = 1138; *p* < 0.001), interaction (F_8,30_ = 60.64; *p* < 0.001) (Table 7).

**Table 7:** Results of a two-way ANOVA of hesperetin-7-*O*-glucuronide concentration in plasma, measured by UHPLC-ESI-HRMS

Independent variables	F-value	*p*-value
Time effect	F_4,30_ = 1138	*p* < 0.001
Treatment effect	F_2,30_ = 229.3	*p* < 0.001
Interaction	F_8,30_ = 60.64	*p* < 0.001

UHPLC-ESI-HRMS: ultra-high-performance liquid chromatography-electrospray ionization-high resolution mass spectrometry

All three experimental groups showed a peak plasma concentration of hesperetin-7-*O*-glucuronide at 4 h post-treatment. Hesperetin-7-*O*-glucuronide plasma AUC analysis shows significantly higher values in the BPFencap group and significantly lower values in the BPFmicro group compared to standard BPF (one-way ANOVA results: F_2,15_=103.9; *p* < 0.001.

## Discussion

The results obtained in this experimental work highlighted that the UHPLC-MS/MS analysis, at all-time points, showed significantly increased plasma levels of naringin and its metabolites within the BPFencap group when compared to the BPF and BPFmicro groups. BPFmicro markedly increased bioavailability, but this was temporary, pointing to a stability problem. The bioavailability and stability of metabolites are significantly improved over time by the new BPFencap formulation.

This study uses UHPLC-ESI-HRMS to determine bioavailability in rat plasma for BPF (standard and new formulations: micronized BPF alone or combined with micronized albedo fibers at 50/50 v/v). The bioavailability study included the presence of phase II conjugated flavanone metabolites at different post-dosing intervals. Circulating levels of these metabolites are a consequence of intestinal cleavage of the juice’s glycoside units, and the following conjugation of flavanone aglycones to glucuronide and sulfate molecules by phase II enzymes. Therefore, investigating the bioactivities of naringenin sulfates and glucuronides, especially the sulfates, is crucial because they might affect how effective naringenin and naringin are in the body [28]. Naringin undergoes quick absorption and metabolism, yielding its aglycone and naringenin glucuronide [32, 33]. Hesperitin-glucuronate rapidly received the neohesperidin. Intestinal bacteria enzymes generally hydrolyze naringin and neohesperidin in the gastrointestinal tract; their aglycones are then absorbed and conjugated. Plasma mainly contains naringenin and hesperitin glucuronide/sulfatase conjugates, with minor free aglycones. The characterization of metabolites and their functions is critical to understanding how BPFs behave in nutraceutical formulations.

Consequently, this study examined the effect of micronization and the combined effect of micronization and fibers on the bioavailability of various bioactive compounds extracted from “pastazzo,” the waste of *Citrus bergamia*. Indeed, dietary fiber is a polysaccharide characterized by being widely available, inexpensive, stable, safe, and non-toxic. Health-boosting functional food ingredients from natural and microbiological sources are becoming more popular. However, using the functional properties of these unstable constituents is difficult. Dietary fiber microencapsulation technology is a promising solution, offering convenient handling and effective protection of encapsulated materials [34]. Bioavailability studies comparing standard BPF to BPFmicro and BPFencap showed significantly increased secondary metabolite bioavailability, likely due to formulation differences. Analysis showed significant differences in naringenin plasma concentration over time between BPFmicro, BPFencap, and standard BPF, with BPFencap exhibiting significantly higher AUC values. Compared to standard BPF and BPFencap, the BPFmicro group had the lowest AUC and significantly lower total concentration.

Previous studies demonstrated the human gut microbiome’s role in converting naringin to naringenin, supporting the idea that fibers increase bioactive compound bioavailability [32]. Naringenin metabolism, as reported by several studies (in vivo and in vitro), primarily results in 5- and/or 4′-glucuronides (via UGT), which are then excreted in the urine of humans and rats [30, 35]. Naringenin’s poor bioavailability may be because its glucuronides, excreted in bile and the gut, undergo enterohepatic cycling via β-glucuronidase and reabsorption, leading to extensive first-pass metabolism [36]. Furthermore, low plasma concentrations of naringenin were found after oral administration and/or the consumption of citrus juices; its lipophilic nature suggests accumulation in tissues like the liver and intestines [37]. Consequently, naringenin’s biological and pharmacological actions are strongly linked to its glucuronidation in the liver and intestines. Hydrolysis with β-glucuronidase is frequently employed in pharmacokinetic investigations of naringin or naringenin due to the recognition of glucuronide and sulphate conjugates of flavonoids as precursors of bioactive metabolites [38]. Specifically, we measured naringenin-4-*O*-glucuronide and naringenin-5-*O*-glucuronide bioavailability, finding that BPFmicro and BPFencap groups showed significantly greater naringenin-4-*O*-glucuronide AUC than the BPF group across time points. Despite previous trends, the BPFencap group showed a considerably higher naringenin-5-*O*-glucuronide plasma concentration than the BPF standard and BPFmicro groups. AUC analysis shows that the BPFencap group experienced a significantly greater increase in naringenin-5-*O*-glucuronide than the BPF group over time. Standard BPF and BPFencap had significantly higher AUCs than the BPFmicro group.

The results obtained suggest that BPF micronization and, in a much more pronounced and stable manner, microinpasulation of micronized BPF into micronized fibers could improve the bioavailability of naringenin and secondary metabolites, precursors of bioactive compounds endowed with the remarkable beneficial properties listed above.

Enhancing polyphenol stability and bioavailability through BPF encapsulation is a strategic approach. A protective wall material encapsulates polyphenols, shielding them from degradation. Polyphenols are shielded by this coating from harsh digestion, reaching the intestine intact [39].

Indeed, evidence has shown that association with fiber may play a key and predominant role in improving inflammation-related dysfunction, hyperlipidemia, and oxidative stress [13]. In addition, we evaluated the secondary metabolites of hesperetin, highlighting that BPFencap showed significantly higher plasma hesperetin-5-*O*-glucuronide concentrations than standard BPF and BPFmicro at all time points. Despite being less pronounced, the BPFmicro group exhibited significantly elevated metabolite levels than the BPF group at every measured time point. AUC analysis showed significantly higher hesperetin-5-*O*-glucuronide levels in BPFmicro and BPFencap groups than in the BPF group. BPFencap levels were significantly higher than BPFmicro levels.

Results from a study suggested the need to improve glucuronide bioavailability, highlighting the Photoprotective Agent Arising from Flavonoid Metabolism in Human Skin Fibroblasts. The study showed that hesperetin glucuronides (hesperetin-5-*O*-glucuronide and hesperetin-7-*O*-glucuronide), but not hesperetin, protect against UV-A-induced necrotic cell death [40].

Consistent with hesperetin-5-*O*-glucuronide, BPFencap showed significantly higher plasma concentrations of hesperetin-7-*O*-glucuronide than BPF and BPFmicro groups across all time points. Compared to standard BPF, hesperetin-7-*O*-glucuronide plasma AUC analysis revealed significantly higher values in the BPFencap group and significantly lower values in the BPFmicro group. The clinical significance of this result is substantial, given naringin 7-*O*-glucuronide’s diverse activities observed in multiple studies. Indeed, adult ovariectomized rats showed improved bone loss prevention with hesperetin-7-*O*-glucuronide, which has greater bioavailability than hesperidin [41].

Additionally, hesperetin-7-*O*-glucuronide plays a role in osteoblast differentiation [42] hesperetin-7-*O*-glucuronide, unlike hesperetin-3′-O-glucuronide, shows hypotensive, vasodilatory, and anti-inflammatory effects like hesperetin, improving hypertension and endothelial dysfunction [43].

This research provides the basis for further preclinical and clinical studies assessing the absorption, distribution, and nutraceutical efficacy of the new formulation compared to the standard BPF in counteracting multi-organ dysfunctions mainly due to metabolic imbalances and gut microbiota alterations.

Indeed, rat bioavailability significantly correlated with human bioavailability. Assessing oral bioavailability is crucial for determining the potential therapeutic applications of nutraceuticals. Although human clinical trials provide the most accurate oral bioavailability data, the expense, safety, and ethical considerations frequently present major obstacles. Animal studies offer data on oral bioavailability, showing the fraction of a dose reaching the bloodstream, but not the process itself [44].

Specifically, the in vivo animal model provides a living system that mimics the complete dynamic physiological and physiochemical processes involved in the absorption, distribution, metabolism, and excretion of orally ingested nutraceuticals. Despite an incomplete understanding of the pharmacokinetic mechanism, analyzing in vivo serum profiles provides useful information regarding oral bioavailability and metabolite profiles following ingestion [44].

## Conclusions

In conclusion, based on the existing evidence in the literature on the key role exerted by BPF metabolites on the prevention of oxidative stress, inflammation, and multiorgan dysfunction, our study demonstrates, for the first time, the presence of the above metabolites in the plasma of rats treated with the different formulations of BPF. Notably, the recent circular economy paradigm has promoted the citrus fruit waste valorization, and our innovative formulation uses “pastazzo,” a byproduct of bergamot industrial processing. Therefore, we have achieved and characterized a new formulation, more bioavailable and able to significantly improve sustainability via eco-friendly manufacturing and green technology.

More impressively, the innovative formulation techniques substantially enhance the absorption of these metabolites, overcoming the low bioavailability of polyphenols.

However, although BPFmicro enhanced bioavailability considerably, the improvement wasn’t sustained, highlighting a formulation stability issue. This problem has been overcome by the development of the new formulation in which the BPFmicro has been encapsulated through hybrid phospholipid systems with citrus albedo fibers (BPFencap), which has been shown to significantly improve the bioavailability of the different metabolites, maintaining it stable over time, as confirmed by the different AUC curves.

Consequently, it could be assumed that BPFencap may be able to maintain, in a more stable way, the nutraceutical efficacy.

## Electronic supplementary material

Below is the link to the electronic supplementary material.

Supplementary Material 1

Supplementary Material 2

Supplementary Material 3

Supplementary Material 4

Supplementary Material 5

## Data Availability

Data is provided within the manuscript or supplementary information files.

## Abbreviations

AUC: Area Under Curve
BMF: Albedo and pulp-derived micronized fibers
BPF: Bergamot polyphenols fraction
BPFencap: Bergamot polyphenols fraction encapsulation
BPFmicro: Bergamot polyphenols fraction micronized
EDTA: Ethylenediaminetetraacetic acid
HESI: Heated electrospray ionization
FWHM: Full width at half maximum
LC/MS: Liquid chromatography-mass spectrometry
LDL: Low-density lipoprotein
LOD: Lower limit of detection
LOQ: Lower limit of quantification
MCS: Microencapsulation citrus system
Milli-Q: Ultrapure water systems
MS/MS: Tandem mass spectrometry
RDA: Retro-Diels-Alder
SCFA: Short-chain fatty Acid
SPE: Solid phase extraction
UGT: Glucuronosyltransferases
UHPLC-MS/MS: Ultra-high performance liquid chromatography-tandem mass spectrometry
UHPLC-ESI-HRMS: Ultra-high-performance liquid chromatography-electrospray ionization-high resolution mass spectrometry

## References

[1] Salmerón-Manzano E, Garrido-Cardenas JA, Manzano-Agugliaro F. Worldwide research trends on medicinal plants. Int J Environ Res Public Health. 2020;12:3376. 10.3390/ijerph17103376 PMC7277765 32408690

[2] Pawase PA, Goswami C, Shams R, Pandey VK, Tripathi A, Rustagi S, et al. A conceptual review on classification, extraction, bioactive potential, and role of phytochemicals in human health. Future Foods. 2024;9:100313.

[3] Hasnat H, Shompa SA, Islam MM, Alam S, Richi FT, Emon NU, et al. Flavonoids: A treasure house of prospective Pharmacological potentials. Heliyon. 2024;10:e27533. 38496846 10.1016/j.heliyon.2024.e27533 PMC10944245

[4] Addi M, Elbouzidi A, Abid M, Tungmunnithum D, Elamrani A, Hano C. An overview of bioactive flavonoids from *Citrus* fruits. Appl Sci. 2022;12:29.

[5] Maruca G, Laghetti G, Mafrica R, Turiano D, Hammeret K. The fascinating history of Bergamot (Citrus Bergamia Risso & Poiteau), the Exclusive Essence of Calabria: A Review. J Environ Sci Eng. 2017;6:22–30.

[6] Suri S, Singh A, Nema PK. Current applications of citrus fruit processing waste: A scientific outlook. Appl Food Res. 2022;2:100050.

[7] Cardamone A, Coppoletta AR, Macrì R, Nucera S, Ruga S, Scarano F, et al. Targeting leptin/CCL3-CCL4 axes in NAFLD/MAFLD: A novel role for BPF in counteracting thalamic inflammation and white matter degeneration. Pharmacol Res. 2024;209:107417. 39276957 10.1016/j.phrs.2024.107417

[8] Mollace R, Macrì R, Tavernese A, Gliozzi M, Musolino V, Carresi C, et al. Comparative effect of Bergamot polyphenolic fraction and red yeast rice extract in rats fed a hyperlipidemic diet: role of antioxidant properties and PCSK9 expression. Nutrient. 2022;14:477. 10.3390/nu14030477 PMC8840352 35276836

[9] Musolino V, Gliozzi M, Nucera S, Carresi C, Maiuolo J, Mollace R, et al. The effect of Bergamot polyphenolicfraction on lipid transfer protein system and vascular oxidative stress in a rat model of hyperlipemia. Lipids Health Dis. 2019;18:115. 31101130 10.1186/s12944-019-1061-0 PMC6525455

[10] Maiuolo J, Carresi C, Gliozzi M, Musolino V, Scarano F, Coppoletta AR, et al. Effects of Bergamot polyphenols on mitochondrial dysfunction and sarcoplasmic reticulum stress in diabetic cardiomyopathy. Nutrients. 2021;13:2476. 34371986 10.3390/nu13072476 PMC8308586

[11] Russo C, Lombardo GE, Bruschetta G, Rapisarda A, Maugeri A, Navarra M. Bergamot byproducts: A sustainable source to counteract inflammation. Nutrients. 2024;16:259. 38257152 10.3390/nu16020259 PMC10819577

[12] Musolino V, Gliozzi M, Bombardelli E, Nucera S, Carresi C, Maiuolo J, et al. The synergistic effect of *Citrus bergamia* and *Cynara cardunculus* extracts on vascular inflammation and oxidative stress in non-alcoholic fatty liver disease. J Tradit Complement Med. 2020;10:268–74. 32670822 10.1016/j.jtcme.2020.02.004 PMC7340872

[13] Mollace R, Macrì R, Nicita M, Musolino V, Gliozzi M, Carresi C, et al. Bergamot polyphenolic extract combined with albedo and pulp fibers counteracts changes in gut microbiota associated with High-Fat diet: implications for lipoprotein size Re-Arrangement. Int J Mol Sci. 2023;24:12967. 37629146 10.3390/ijms241612967 PMC10454550

[14] Benameur T, Porro C, Twfieg ME, Benameur N, Panaro MA, Filannino FM, et al. Emerging paradigms in inflammatory disease management: exploring bioactive compounds and the gut microbiota. Brain Sci. 2023;13:1226. 37626582 10.3390/brainsci13081226 PMC10452544

[15] González-Aguilar GA, Blancas-Benítez FJ, Sáyago-Ayerdi SG. Polyphenols associated with dietary fibers in plant foods: molecular interactions and bioaccessibility. Curr Opin Food Sci. 2017;13:84–8.

[16] Jakobek L, Matić P. Non-covalent dietary fiber - Polyphenol interactions and their influence on polyphenol bioaccessibility. Trends Food Sci Technol. 2019;83:235–47.

[17] Thebaudin JY, Lefebvre AC, Harrington M, Bourgeois CM. Dietary fibre: nutritional and technological interest. Trends Food Sci Technol. 1997;8:41–8.

[18] Sefrin Speroni C, Rigo Guerra D, Beutinger Bender AB, Stiebe J, Ballus CA, Picolli da Silva L, et al. Micronization increases the bioaccessibility of polyphenols from granulometrically separated Olive pomace fractions. Food Chem. 2021;344:128689. 33277120 10.1016/j.foodchem.2020.128689

[19] Tomás-Navarro M, Vallejo F, Borrego F, Tomás-Barberán FA. Encapsulation and micronization effectively improve orange beverage Flavanone bioavailability in humans. J Agric Food Chem. 2014;62:9458–62. 25200135 10.1021/jf502933v

[20] Nemzer BV, Al-Taher F, Kalita D, Yashin AY, Yashin YI. Health-Improving effects of polyphenols on the human intestinal microbiota: A review. Int J Mol Sci. 2025;26:1335. 39941107 10.3390/ijms26031335 PMC11818678

[21] Mollace V, Scicchitano M, Paone S, Casale F, Calandruccio C, Gliozzi M, et al. Hypoglycemic and hypolipemic effects of a new lecithin formulation of Bergamot polyphenolic fraction: A double blind, randomized, Placebo- controlled study. Endocr Metab Immune Disord Drug Targets. 2019;19:136–43. 30501605 10.2174/1871530319666181203151513

[22] Musolino V, Gliozzi M, Scarano F, Bosco F, Scicchitano M, Nucera S, et al. Bergamot polyphenols improve dyslipidemia and pathophysiological features in a mouse model of Non-Alcoholic fatty liver disease. Sci Rep. 2020;10:2565. 32054943 10.1038/s41598-020-59485-3 PMC7018973

[23] Ilari S, Lauro F, Giancotti LA, Malafoglia V, Dagostino C, Gliozzi M, et al. The protective effect of Bergamot polyphenolic fraction (BPF) on Chemotherapy-Induced neuropathic pain. Pharmaceuticals. 2021;14:975. 34681199 10.3390/ph14100975 PMC8540578

[24] Parafati M, Lascala A, Morittu VM, Trimboli F, Rizzuto A, Brunelli E, et al. Bergamot polyphenol fraction prevents nonalcoholic fatty liver disease via stimulation of lipophagy in cafeteria diet-induced rat model of metabolic syndrome. J Nutr Biochem. 2015;26:938–48. 26025327 10.1016/j.jnutbio.2015.03.008

[25] Frisina M, Bonacci S, Oliverio M, Nardi M, Vatrano TP, Procopio A. Storage effects on bioactive phenols in Calabrian monovarietal extra Virgin Olive oils based on the EFSA health claim. Foods. 2023;12:3799. 37893692 10.3390/foods12203799 PMC10606493

[26] Zhang J, Brodbelt JS. Screening flavonoid metabolites of naringin and Narirutin in urine after human consumption of grapefruit juice by LC-MS and LC-MS/MS. Analyst. 2004;129:1227–33. 15565223 10.1039/b412577k

[27] Zeng X, Su W, Zheng Y, Liu H, Li P, Zhang W, et al. UFLC-Q-TOF-MS/MS-Based screening and identification of flavonoids and derived metabolites in human urine after oral administration of Exocarpium citri grandis extract. Molecules. 2018;23:895. 29649170 10.3390/molecules23040895 PMC6017061

[28] Durmus N, Gulsunoglu-Konuskan Z, Kilic-Akyilmaz M, Recovery. Bioactivity, and utilization of bioactive phenolic compounds in *Citrus* Peel. Food Sci Nutr. 2024;12:9974–97. 39723030 10.1002/fsn3.4570 PMC11666827

[29] Liu M, Zou W, Yang C, Peng W, Su W. Metabolism and excretion studies of oral administered naringin, a putative antitussive, in rats and dogs. Biopharm Drug Dispos. 2012;33:123–34. 22374702 10.1002/bdd.1775

[30] Xu F, Liu Y, Zhang Z, Yang C, Tian Y. Quasi-MSn identification of Flavanone 7-glycoside isomers in Da Chengqi Tang by high performance liquid chromatography-tandem mass spectrometry. Chin Med. 2009;4:15. 19630957 10.1186/1749-8546-4-15 PMC2722651

[31] Ji Z, Deng W, Chen D, Liu Z, Shen Y, Dai J, et al. Recent Understanding of the mechanisms of the biological activities of hesperidin and Hesperetin and their therapeutic effects on diseases. Heliyon. 2024;10:26862. 10.1016/j.heliyon.2024.e26862 PMC10937595 38486739

[32] Macrì R, Mollace R, Serra M, Scarano F, Ritorto G, Ussia S, et al. Nutritional and nutraceutical support to the failing myocardium: A possible way of potentiating the current treatment of heart failure. Int J Mol Sci. 2024;25:12232. 39596298 10.3390/ijms252212232 PMC11594499

[33] Yuan J, Wei F, Luo X, Zhang M, Qiao R, Zhong M, et al. Multi-Component comparative pharmacokinetics in rats after oral administration of *Fructus aurantii* extract, naringin, neohesperidin, and Naringin-Neohesperidin. Front Pharmacol. 2020;11:933. 32636752 10.3389/fphar.2020.00933 PMC7319089

[34] Zhang Y, Amin K, Zhang Q, Yu Z, Jing W, Wang Z, et al. The application of dietary fibre as microcapsule wall material in food processing. Food Chem. 2025;463:141195. 39276558 10.1016/j.foodchem.2024.141195

[35] Zeng X, Zheng Y, He Y, Zhang J, Peng W, Su W. Microbial metabolism of naringin and the impact on antioxidant capacity. Nutrients. 2022;14:3765. 36145140 10.3390/nu14183765 PMC9502552

[36] Manach C, Scalbert A, Morand C, Rémésy C, Jiménez L. Polyphenols: food sources and bioavailability. Am J Clin Nutr. 2004;79:727–47. 15113710 10.1093/ajcn/79.5.727

[37] Erlund I. Review of the flavonoids quercetin, hesperetin, and naringenin. Dietary sources, bioactivities, bioavailability, and epidemiology. Nutr Res. 2004;24:851–74.

[38] Matsumoto T, Kaneko A, Koseki J, Matsubara Y, Aiba S, Yamasaki K. Pharmacokinetic study of bioactive flavonoids in the traditional Japanese medicine Keigairengyoto exerting antibacterial effects against *Staphylococcus aureus*. Int J Mol Sci. 2018;19:328. 29360768 10.3390/ijms19020328 PMC5855550

[39] Ali Redha A, Kodikara C, Cozzolino D. Does encapsulation improve the bioavailability of polyphenols in humans?? A concise review based on in vivo humans? studies. Nutrients. 2024;16:3625. 39519458 10.3390/nu16213625 PMC11547751

[40] Proteggente AR, Basu-Modak S, Kuhnle G, Gordon MJ, Youdim K, Tyrrell R, et al. Hesperetin glucuronide, a photoprotective agent arising from flavonoid metabolism in human skin fibroblasts. Photochem Photobiol. 2003;78:256–61. 14556312 10.1562/0031-8655(2003)078<0256:hgapaa>2.0.co;2

[41] Habauzit V, Nielsen IL, Gil-Izquierdo A, Trzeciakiewicz A, Morand C, Chee W, et al. Increased bioavailability of hesperetin-7-glucoside compared with hesperidin results in more efficient prevention of bone loss in adult ovariectomised rats. Br J Nutr. 2009;102:976–84. 19393110 10.1017/S0007114509338830

[42] Trzeciakiewicz A, Habauzit V, Mercier S, Barron D, Urpi-Sarda M, Manach C, et al. Molecular mechanism of Hesperetin-7-*O*-glucuronide, the main Circulating metabolite of hesperidin, involved in osteoblast differentiation. J Agric Food Chem. 2010;58:668–75. 19921838 10.1021/jf902680n

[43] Yamamoto M, Jokura H, Hashizume K, Ominami H, Shibuya Y, Suzuki A, et al. Hesperidin metabolite hesperetin-7-O-glucuronide, but not hesperetin-3’-O-glucuronide, exerts hypotensive, vasodilatory, and anti-inflammatory activities. Food Funct. 2013;4:1346–51. 23831969 10.1039/c3fo60030k

[44] Ting Y, Zhao Q, Xia C, Huang Q. Using in vitro and in vivo models to evaluate the oral bioavailability of nutraceuticals. J Agric Food Chem. 2015;63:1332–8. 10.1039/c3fo60030k. 25615514 10.1021/jf5047464

