# Supplementary material for: The second life of Citrus bergamia: bioavailability analysis of a new formulation using waste-based microencapsulation as a valuable source of bioactive compounds
Source: Pharmacol Rep. 2025 Jul 25;77(5):1400–14. doi: 10.1007/s43440-025-00758-x (PMC12443862; doi:10.1007/s43440-025-00758-x)

SAMPLE REPORT

Acquisition Date/Time	14-Oct-22 09:27:10
Acquisition Method	Flavonoids ca. on Naringin_March22
Chromera Version	4.2.0.6415
Dilution Factor	21
Report Date/Time	19-Oct-22 10:59:44
Sample Name	Bergamot Fiber Lot. 1402-22
Vial Number	53

Bergamot Fiber Lot. 1402-22 : 284:10:400:10 : 1

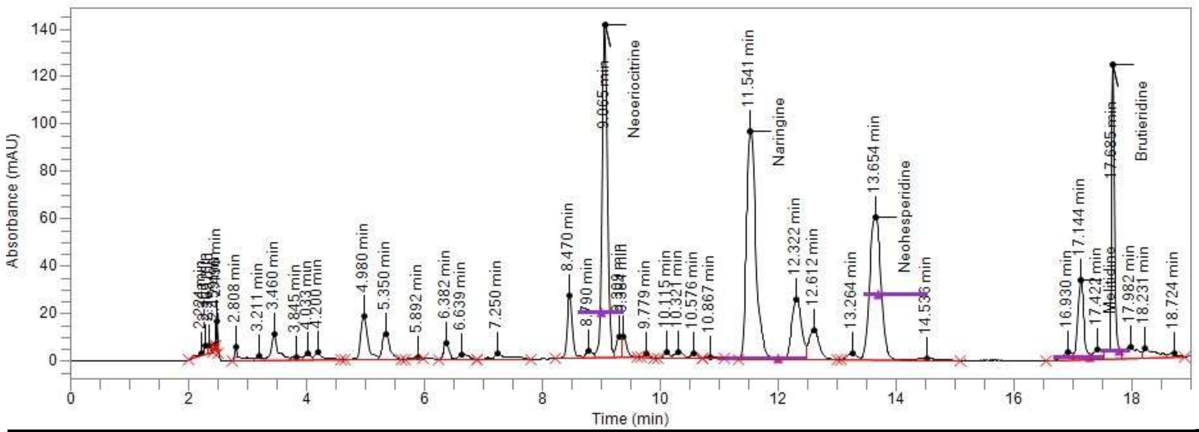

Peak #	Time	Component Name	Area	Height	Final Amount	Units
37	17.685	Brutieridine	460,428.7	124,257.2	2,286.9272	ppm
35	17.144	Melitidine	240,632.0	33,864.1	1,233.3295	ppm
28	11.541	Naringine	1,064,965.6	96,201.0	5,184.8012	ppm
20	9.065	Neoeriocitrine	900,833.9	140,543.2	4,398.0290	ppm
32	13.654	Neohesperidine	810,831.1	60,168.6	3,966.5970	ppm
**Total**			3,477,691.3		17,069.6840	

Figure S1: Chromatogram obtained from citrus albedo fibers using ultra high liquid chromatography.

SAMPLE REPORT

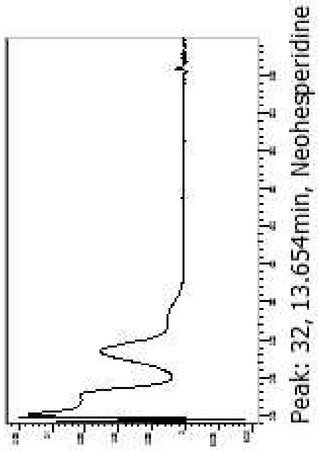

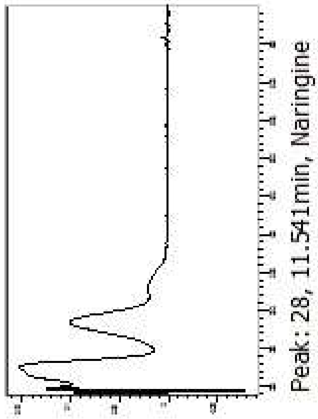

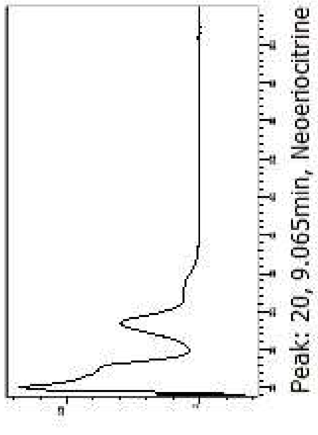

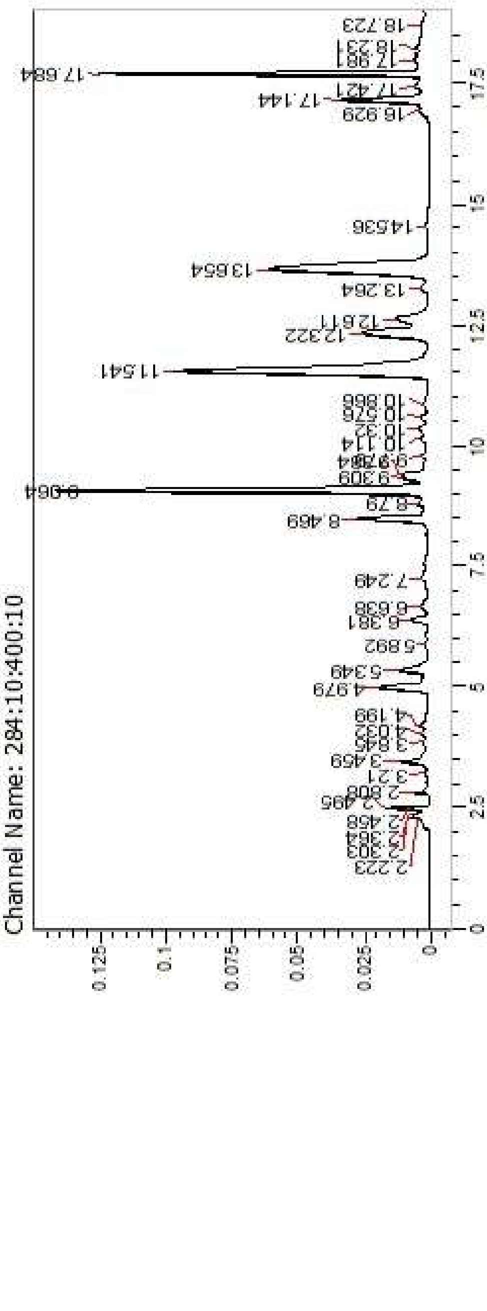

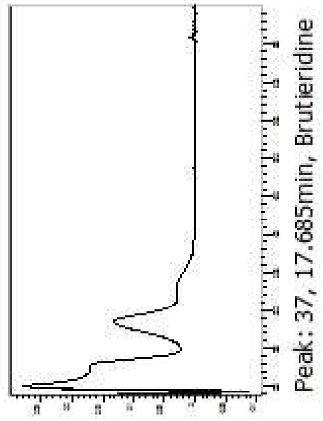

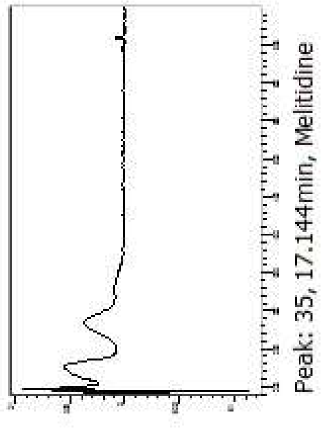

Supplement: Supplementary file 1 — Supplementary Material 1 [file 43440_2025_758_MOESM1_ESM.pdf]
