# Supplementary material for: The second life of Citrus bergamia: bioavailability analysis of a new formulation using waste-based microencapsulation as a valuable source of bioactive compounds
Source: Pharmacol Rep. 2025 Jul 25;77(5):1400–14. doi: 10.1007/s43440-025-00758-x (PMC12443862; doi:10.1007/s43440-025-00758-x)
Supplement: Supplementary file 3 — Supplementary Material 3 [file 43440_2025_758_MOESM3_ESM.pdf]

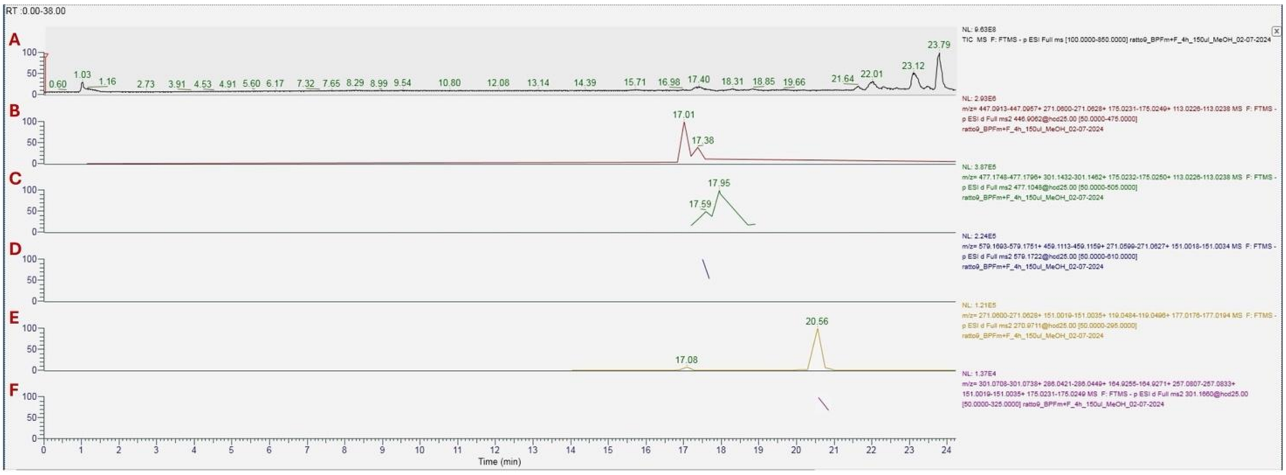

Figure S3: Full Scan A) chromatogram obtained by UHPLC-ESI-HRMS $[M-H]^-$ of a representative rat plasma sample and metabolite ion chromatograms in a representative rat plasma sample B) naringin-5-*O*-glucuronide and naringin-4-*O*-glucuronide, C) hesperetin-7-*O*-glucuronide and hesperetin-5-*O*-glucuronide D) naringin E) naringenin F) hesperetin.
