# Supplementary material for: The second life of Citrus bergamia: bioavailability analysis of a new formulation using waste-based microencapsulation as a valuable source of bioactive compounds
Source: Pharmacol Rep. 2025 Jul 25;77(5):1400–14. doi: 10.1007/s43440-025-00758-x (PMC12443862; doi:10.1007/s43440-025-00758-x)
Supplement: Supplementary file 4 — Supplementary Material 4 [file 43440_2025_758_MOESM4_ESM.pdf]

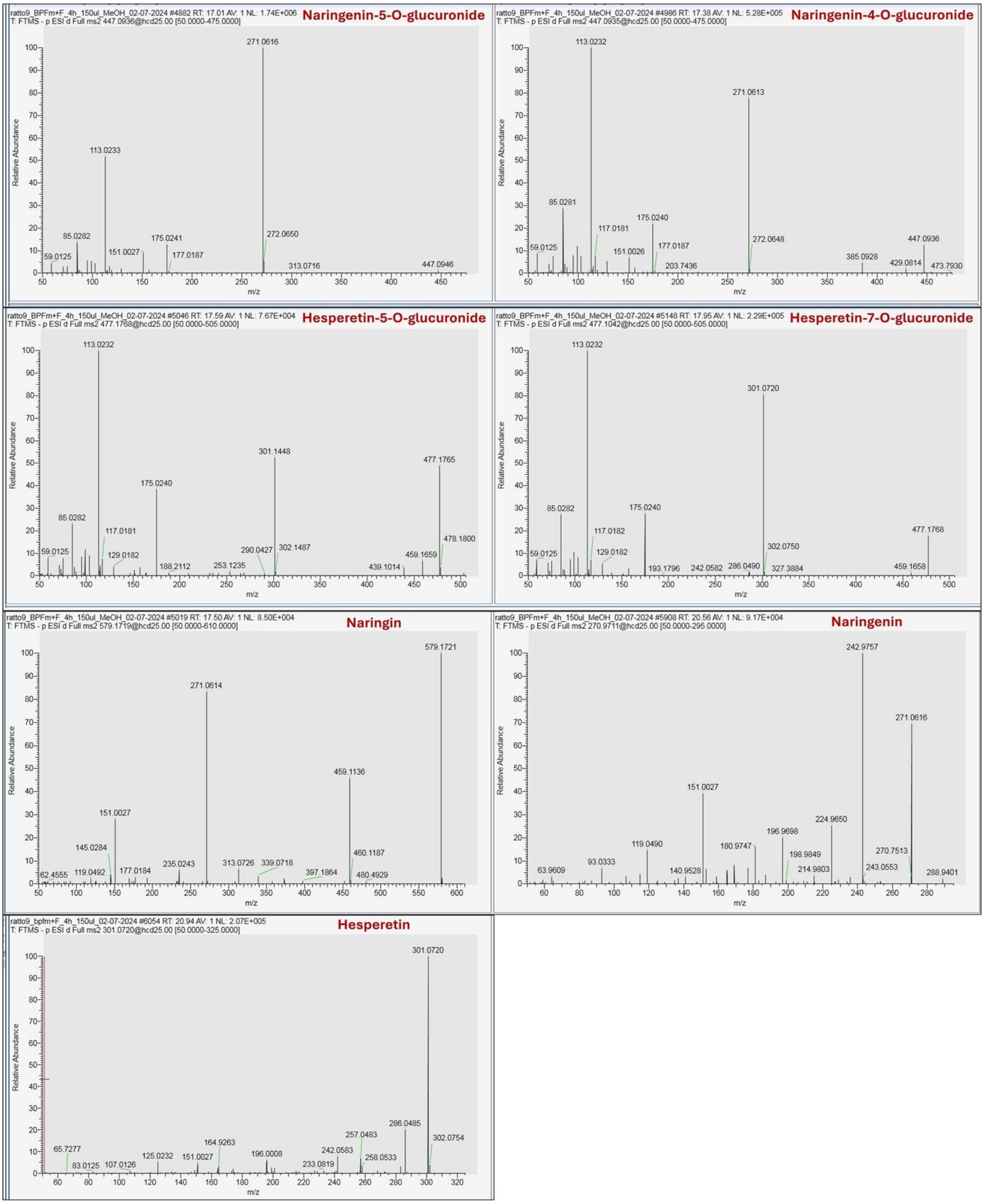

Figure S4: MSMS spectra obtained by UHPLC-ESI-HRMS for the [M-H]- of a representative rat plasma sample after solid phase extraction. Naringin-5-*O*-glucuronide, naringin-4-*O*-glucuronide, hesperetin-7-*O*-glucuronide, hesperetin-5-*O*-glucuronide, naringin, naringenin, hesperetin.
