# Supplementary material for: The second life of Citrus bergamia: bioavailability analysis of a new formulation using waste-based microencapsulation as a valuable source of bioactive compounds
Source: Pharmacol Rep. 2025 Jul 25;77(5):1400–14. doi: 10.1007/s43440-025-00758-x (PMC12443862; doi:10.1007/s43440-025-00758-x)
Supplement: Supplementary file 5 — Supplementary Material 5 [file 43440_2025_758_MOESM5_ESM.pdf]

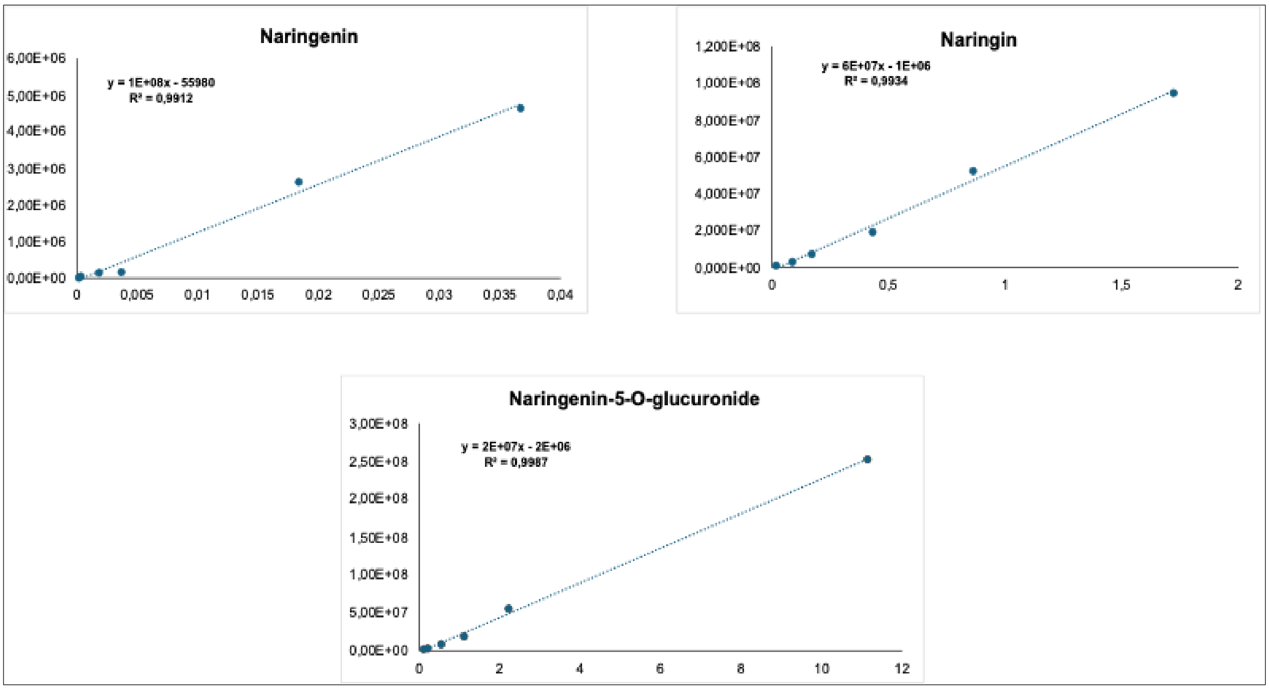

Figure S5: Calibration curve of corresponding reference standards. The concentrations of naringenin (271.0616 *m/z*) were obtained using the calibration curve of the corresponding reference standards (r^2=0.9912) in a range between 0.00018µM and 0.03673 µM. The concentration of naringin (579.1721 *m/z)* were obtained using the calibration curve of the corresponding reference standards (r^2=0.9934) in a range between 0.01722µM -1.72253µM. naringin-*5-O*-glucuronide (447.0946 *m/z*), hesperetin-7-*O*-glucuronide (477.1768 *m/z*), naringenin-4-*O*-glucuronide (447.0936 *m/z*), hesperetin-5-*O*-glucuronide (477.1765 *m/z*) were obtained respect to a calibration curve of naringenin-5-O-glucuronide (447.0946 *m/z*) reference standards (r^2=0.9987) in a range between 0.11150 µM - 11.15075 µM.
